# Interplay between human nucleolar GNL1 and RPS20 is critical to modulate cell proliferation

**DOI:** 10.1038/s41598-018-29802-y

**Published:** 2018-07-30

**Authors:** Rehna Krishnan, Neelima Boddapati, Sundarasamy Mahalingam

**Affiliations:** Laboratory of Molecular Virology and Cell Biology, Department of Biotechnology, Bhupat and Jyoti Mehta School of Biosciences, Indian Institute of Technology-Madras, Chennai, 600 036 India

## Abstract

Human Guanine nucleotide binding protein like 1 (GNL1) belongs to HSR1_MMR1 subfamily of nucleolar GTPases. Here, we report for the first time that GNL1 promotes cell cycle and proliferation by inducing hyperphosphorylation of retinoblastoma protein. Using yeast two-hybrid screening, Ribosomal protein S20 (RPS20) was identified as a functional interacting partner of GNL1. Results from GST pull-down and co-immunoprecipitation assays confirmed that interaction between GNL1 and RPS20 was specific. Further, GNL1 induced cell proliferation was altered upon knockdown of RPS20 suggesting its critical role in GNL1 function. Interestingly, cell proliferation was significantly impaired upon expression of RPS20 interaction deficient GNL1 mutant suggest that GNL1 interaction with RPS20 is critical for cell growth. Finally, the inverse correlation of GNL1 and RPS20 expression in primary colon and gastric cancers with patient survival strengthen their critical importance during tumorigenesis. Collectively, our data provided evidence that cross-talk between GNL1 and RPS20 is critical to promote cell proliferation.

## Introduction

The YawG/YIqF/HSR1_MMR1 GTP-binding protein subfamily of GTPases is evolutionarily conserved across from prokaryotes to mammals. The members of this family have shown to be involved in ribosomal assembly and ribosomal RNA processing and are characterized by the presence of circular permutation of guanine nucleotide binding motifs^1^. The guanine nucleotide motifs G1-G5 of YawG/YIqF GTPases are arranged in G5-G4-G1-G2-G3 order whereas G1-G2-G3-G4-G5 order in classical GTPases^2^. The four well known members of this family are GNL1, GNL2, GNL3 and GNL3L and the expression levels of all were upregulated in most cancers^3,4^. These GTPases are found to be shuttling between nucleolus, nucleus and cytoplasm^5,6^. Depletion of GNL2, GNL3 and GNL3L has shown to alter G1/S and G2/M cell cycle transition indicates their role in cell cycle regulation^7–9^ but the molecular mechanism yet to be defined.

GNL1 is a putative nucleolar GTPase belonging to YawG/YIqF subfamily but the function remains largely unknown. It encodes 607 amino acids with a molecular mass of 65 kDa and contains basic amino acids rich N-terminus, acidic amino acids rich C-terminus and proline rich-domains. Previous report from our laboratory provided evidence that GNL1 harbors a novel arginine/lysine-rich nuclear/nucleolar localization signal and localized in different subcellular compartments in cell cycle dependent manner^10^. The presence of GTP binding motifs indicate that GNL1 can acts as molecular switch to control its transition between nucleus and cytoplasm (10). GNL1 plays a critical role in liver cell proliferation^11^ and found to be upregulated in bladder and ovarian cancer and in panel of squamous cell carcinoma cell lines^12–14^. However, the function of GNL1 during tumorigenesis remains largely unknown.

Several nucleotide binding proteins have been shown to play critical role in ribosome biogenesis^1^. GNL family of GTPases are known to be involved in rRNA processing and ribosome biogenesis^15^. GNL3L and GNL3 (nucleostemin) are localized in the nucleolus and modulate ribosomal as well as non-ribosomal pathways^15–21^ to promote cell proliferation. Several reports suggest that a functional interaction of GNL family members with large and small ribosomal proteins^7,8,20^ but the functional consequences of these interactions are poorly understood. Studies are warranted to understand whether GNL1 participates in ribosomal biogenesis or has some non-ribosomal functions to regulate cell proliferation during tumorigenesis.

In the present investigation, using yeast two-hybrid assay, ribosomal protein S20 (RPS20) was identified as a novel functional interacting partner of GNL1. Furthermore, our results suggest that GNL1 and RPS20 promotes phosphorylation of retinoblastoma protein (Rb) which in-turn modulate G1/S phase of the cell division cycle. In addition, the interplay between GNL1 and RPS20 is critical to promote the cell proliferation and survival during tumorigenesis.

## Results

### GNL1 promotes cell proliferation

**Figure 1:**
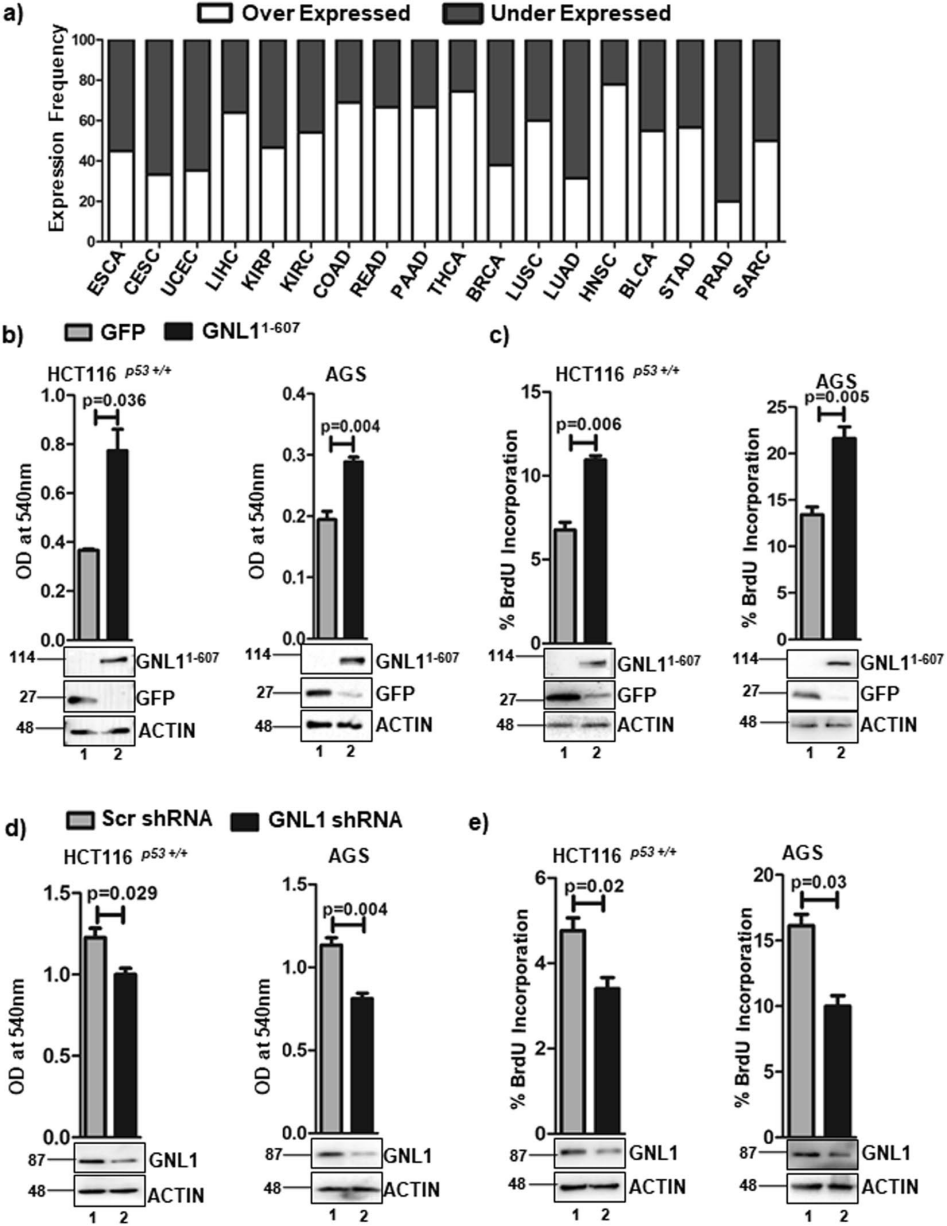
GNL1 promotes cell proliferation. (**a**) Expression of GNL1 in primary cancer tissues from BioXpress database (https://hive.biochemistry.gwu.edu/tools/bioxpress). (**b**) MTT and (**c**) BrdU incorporation assay were carried out after 48 hours of GNL1^1–607^ or GFP transfection in HCT116^*p53*+/+^ and AGS cells. (**d**) MTT and (**e**) BrdU incorporation assay were carried out after 72 hours of transfection of HCT116^*p53*+/+^ and AGS cells with GNL1 specific shRNA or scrambled shRNA. β-actin was used as loading control. The full-length blots are presented in Supplementary Fig. 6.

GNL1 is an evolutionary conserved nucleolar GTP binding protein belongs to YawG/YIqF subfamily of GTPases. The previous report from our group provided evidence that GNL1 modulates cell division cycle to promote cell proliferation^10^, but the mechanism remains unexplored. To this end, we first analyzed the expression patterns of GNL1 in different cancers with respective normal tissues available in Bio-Xpress database^22^. Results from this analysis suggested that GNL1 expression was upregulated in majority of the cancers (Fig. 1a). Based on GNL1 expression pattern, colorectal and gastric cancer cell line systems were selected to further understand the functional relevance of GNL1 upregulation during tumorigenesis. Towards this, we first determined the cell survival/proliferation by MTT and BrdU incorporation assays upon ectopic expression of GNL1 in colorectal (HCT116^*p53*+/+^) and gastric (AGS) cancer cell lines. Results in Fig. 1b and c indicated that GNL1^1–607^-GFP (here after referred as GNL1^1–607^) increased cell viability and proliferation of both cell lines tested. In contrast, depletion of GNL1 by specific shRNA significantly reduced cell viability and proliferation of HCT116^*p53*+/+^ as well as AGS cell lines (Fig. 1d and e). Ectopic expression and depletion of GNL1 were determined by western blot analysis using anti-GFP and anti-GNL1 antibodies, respectively. Together, these data suggest that GNL1 promotes cell survival and proliferation.

### GNL1 regulates G1/S phase of the cell cycle through Rb phosphorylation

**Figure 2:**
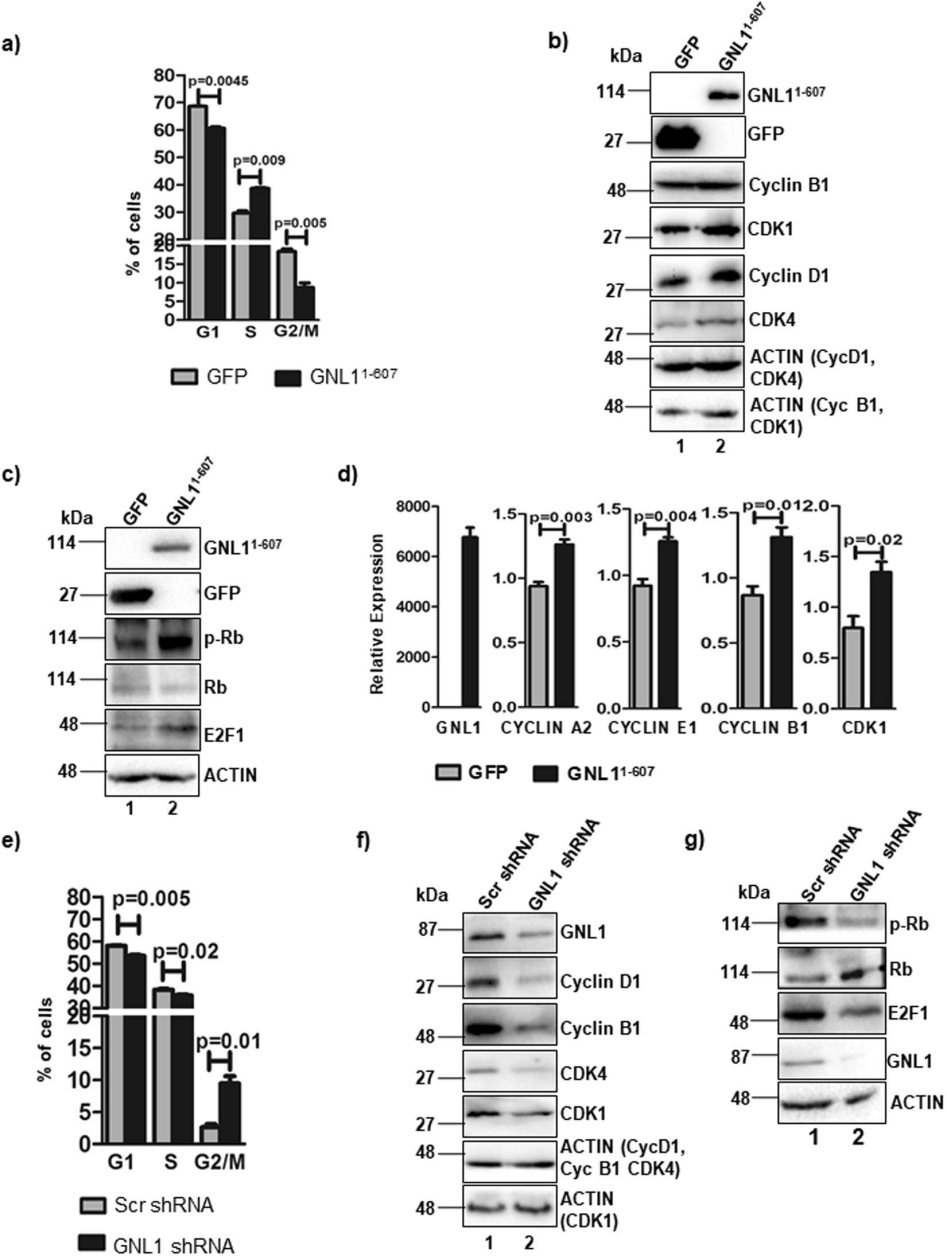
GNL1 modulates cell cycle progression through Rb phosphorylation. (**a**) Cell cycle analysis in HCT116^*p53*+/+^ cells indicated a significant change in G1/S and G2/M phase of the cell cycle upon overexpression of GNL1^1–607^. (**b**) Western blot was performed to analyze the level of cyclin B1, CDK1, cyclin D1 and CDK4 upon GNL1^1–607^ expression. (**c**) Ectopic expression of GNL1^1–607^ increased hyperphosphorylation of Rb at serine 780 and upregulated endogenous E2F1 protein level. The full-length blots are presented in Supplementary Fig. 7. (**d**) The expression levels of cyclin A2, cyclin E1, cyclin B1 and CDK1 were determined by RT-qPCR analysis upon ectopic expression of GNL1^1–607^. (**e**) GNL1 depletion resulted in increased accumulation of HCT116^*p53*+/+^ cells in G2/M phase of cell cycle. Scrambled shRNA was used as control. (**f**) Expression profile of cyclin D1, cyclin B1, CDK4 and CDK1 in HCT116^*p53*+/+^ cells upon GNL1 knockdown. (**g**) The expression of total, phosphorylated Rb and E2F1 protein levels were checked by western blot analysis using anti-phosho Rb (S780), anti-Rb and anti-E2F1 antibodies upon GNL1 knockdown. β-actin was used as loading control. The full-length blots are presented in Supplementary Fig. 8.

Previous reports from others and our group suggest that GNL family of GTPases (GNL2, GNL3 and GNL3L) were known to regulate cell division cycle^6–8,20^ but the role of GNL1 during cell division cycle remain poorly understood. Earlier report suggests that GNL1 might modulate G2/M phase of cell cycle^10^. To further understand the mechanism of GNL1 mediated cell cycle progression, GNL1^1–607^ was ectopically expressed in HCT116^*p53*+/+^ cells and cell cycle analysis was performed. Interestingly, GNL1^1–607^ significantly reduced the accumulation of cells in G1/S and G2/M phases (Fig. 2a). In contrast, more cells were accumulated in ‘S’ phase of the cell cycle (Fig. 2a) which was supported by increased BrdU incorporation (Fig. 1c) upon GNL1^1–607^ expression. Towards dissecting the mechanism by which GNL1 modulates cell cycle, the levels of endogenous cyclins and cyclin-dependent kinase (CDKs) were determined upon GNL1^1–607^ expression. Results in Fig. 2b indicated that cyclin D1-CDK4 (G1/S phase regulator) as well as cyclin B1-CDK1 (G2/M phase regulator) levels were significantly upregulated upon GNL1^1–607^ expression (lane 2). It is well documented that cyclin D1-CDK4/6 mediated hyperphosphorylation of Rb is a critical step in overriding restriction point of G1/S phase transition^23^. Basically, Cyclin D1-CDK4/6 complex mediated phosphorylation of Rb at Serine 780 (pRb^S780^) resulted in release of E2F1 from Rb-E2F1 inhibitory complex to activate the expression of its target genes like cyclin A2, cyclin E1 and cyclin B1 to regulate different stages of cell cycle^24,25^. We next checked the status of Rb phosphorylation at serine 780 upon GNL1^1–607^ expression. Interestingly, the level of pRb^S780^ was increased in GNL1 expressing cells compared to vector transfected cells without altering total Rb protein levels (Fig. 2c, lane 2). Interestingly, E2F1 protein level was also increased upon GNL1^1–607^ expression (Fig. 2c, lane 2). In addition, RT-qPCR analysis reveals that the mRNA levels of cyclin A2, cyclin E1, cyclin B1 and CDK1 were increased in presence of GNL1^1–607^ expression (Fig. 2d) suggest that GNL1 regulates the expression of cyclins and cyclin dependent kinases at transcriptional levels to promote cell cycle progression. To further define the specificity of GNL1 role in cell cycle regulation, endogenous GNL1 level was depleted by specific shRNA and observed that more number of cells were accumulated in the G2/M phase and lesser number of cells in the G1/S phase of cell cycle (Fig. 2e) strengthens the notion that GNL1 regulates cell division cycle.

The observed cell cycle arrest at G2/M phase under GNL1 knockdown condition may be due to the changes in cyclins and CDKs levels. Western blot analysis indicated that the levels of cyclin D1, cyclin B1, CDK4 and CDK1 were significantly downregulated upon GNL1 knockdown (Fig. 2f, lane 2). We next checked the effect of GNL1 knockdown on the status of Rb phosphorylation. Results in Fig. 2g clearly indicate that a significant reduction of pRb^S780^ level and E2F1 protein level in GNL1 depleted cells (Lane 2). Taken together, these results provide evidence that GNL1 modulates the levels of cyclins, CDKs and the status of Rb phosphorylation to promote cell cycle progression.

### RPS20 was identified as a novel functional interacting partner for GNL1

**Figure 3:**
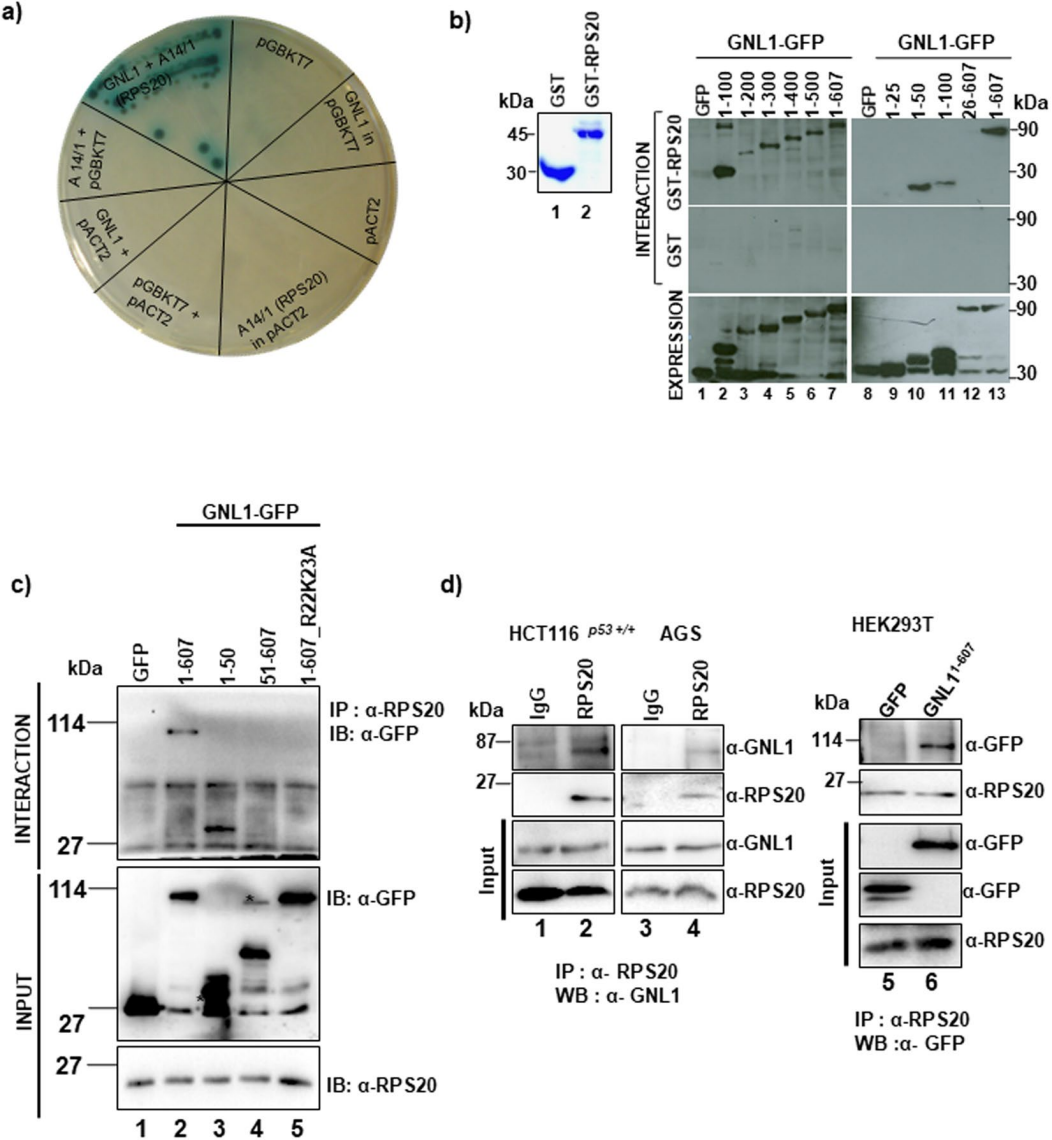
Yeast two-hybrid screen identified RPS20 as a novel interacting partner of GNL1. (**a**) GNL1 and RPS20 interaction was confirmed by transforming A14/1 clone that encodes RPS20^41–119^ into AH109 cells expressing GNL1. (**b**) GNL1^1–50^ is a minimal domain required for GNL1 interaction with RPS20. HEK293T cells were transfected with full length or indicated c-terminal deletion constructs of GNL1^1–607^. GST pull down assay was performed with GST-RPS20 followed by western blotting using anti-GFP antibody (Top Panel). GST was used as negative control (Middle Panel). The expression of GNL1 and its deletion constructs were checked by western blotting with anti-GFP antibody (Bottom Panel). (**c**) HCT116^*p53*+/+^ cells were transfected with full length or indicated variants of GNL1^1–607^. The cell lysates were subjected to co-immunoprecipitation with anti-RPS20 antibody followed by western blotting using anti-GFP antibody. (**d**) HCT116^*p53*+/+^ and AGS cell lysates were subjected to co-immunoprecipitation using anti-RPS20 antibody. The antibody-protein complexes were separated on SDS-12%PAGE followed by western blot analysis with anti-GNL1 antibody. HEK293T cells were transfected with GNL1^1–607^ or control vector. Co-immunoprecipitation was carried out with anti-RPS20 antibody followed by western blotting with anti-GFP antibody. The full-length blots are given in Supplementary Fig. 9.

Towards understanding the mechanism by which GNL1 promotes cell proliferation, we performed yeast two-hybrid assay^26^ to identify the functional interacting partner for GNL1. Since GNL1 is ubiquitously expressed^27^, human leukocyte cDNA library (as HA tag fusion) was used as prey in this interaction assay. Full length GNL1 was cloned into pGBKT7 vector as fusion with c-Myc and used as bait. AH109 yeast cells expressing GNL1 were transformed with leukocyte cDNA library and the positive colonies were selected in high stringency media (SD X-α-Gal/-Ade/-His/-Leu/-Trp) to avoid non-specific interactions as described in Materials and Methods. Total yeast DNA was isolated from blue colored colonies and transformed into *E*. *coli* MC1061 cells. Screening procedure was detailed in Supplementary Fig. S1a. Based on the sequencing of positive clones, seven novel GNL1 interacting partners such as Ribosomal protein S20 (RPS20), Isocitrate dehydrogenase 3 gamma (IDH3G), Filamin A (FLNA), Serpin B1, Poly(rC) binding protein 2 (PCBP2), Microtubule interacting and transport domain containing 1 (MITD1) and Structural maintenance of chromosomes flexible hinge domain containing 1 (SmcHD1) were identified. Members of GNL family were reported to be involved in ribosome biogenesis pathway (1). GNL1 was known to localize in the nucleolus (10) and may have some role in ribosomal biogenesis or cell cycle, so we selected RPS20 for further characterization to understand GNL1 function. Based on the sequencing analysis, we have identified amino acids 41 to 119 of RPS20 (clone A14/1) was required for interaction with GNL1 (Supplementary Fig. S1b). To further confirm the specificity of GNL1-RPS20 interaction, clone A14/1 was transformed into AH109 yeast cells expressing GNL1 and the transformants were selected on SD X-α-Gal/-Ade/-His/-Leu/-Trp agar plates. The blue colored colonies indicated the specific interaction between GNL1 and RPS20 (Fig. 3a). AH109 cells transformed with various indicated plasmids either singly or in combination were used as a negative control to confirm the specificity/integrity of GNL1-RPS20 interaction.

We next mapped the minimal domain in GNL1 required for its interaction with RPS20. Various C-terminal deletion constructs of GNL1^1–607^ were generated as fusion with GFP and expressed in HEK293T cells. GST-RPS20 was expressed in *E*. *coli* BL21DE3 cells and GST-pull down assay was performed as described in Materials and Methods. Glutathione-Sepharose beads bound GST-RPS20 were incubated with HEK293T cell lysates containing equal amounts of GFP, full length or indicated variants of GNL1^1–607^ (Supplementary Fig. S2a) and the bound proteins were analyzed by western blot analysis. Surprisingly, results in Fig. 3b indicate that domain resided within amino acids 1 to 100 of GNL1 is critical for its interaction with RPS20 (lane 1–7). To further identify the minimal domain within GNL1 required for RPS20 interaction, various deletion constructs within amino acids 1 to 100 of GNL1^1–607^ were generated (Supplementary Fig. S2a) and used in GST-pull down assay as described above. The results in Fig. 3b indicate that GNL1 mutant containing amino acids 1 to 50 interacted with RPS20 like GNL1^1–607^. Interestingly, GNL1^1–25^ and GNL1^26–607^ (Fig. 3b, lane 9 and 12) mutants were failed to interact with RPS20 suggests that amino acids 1 to 50 of GNL1 is critical for its interaction with RPS20. GFP and GST were used as negative controls. The integrity of purified GST and GST-RPS20 used for the pull-down assay were shown in Fig. 3b. Lack of interaction between GFP with GST-RPS20 and GST with variants of GNL1^1–607^ indicated the specificity of interaction between GNL1 and RPS20. To define the domain in RPS20 essential for its interaction with GNL1, various truncation mutants of RPS20 were generated as GST fusion (Supplementary Fig. S2b). GST pull down assay was carried out with full length or indicated deletion constructs of GST-RPS20 and HEK293T cell lysates containing GFP or GNL1^1–607^ as described in Materials and Methods. Results in Supplementary Fig. S2c suggest that GNL1^1–607^ interacted with RPS20 full length and the deletion constructs of GST-RPS20 containing amino acids 41 to 60 suggest that amino acids between 41–60 is the minimal domain in RPS20 required for its interaction with GNL1.

Further, co-immunoprecipitation experiments were performed to confirm the interaction between GNL1 and RPS20 is existed in mammalian cells. Towards this, GNL1^1–607^, GNL1^1–50^ and GNL1^51–607^ were transiently expressed in HCT116^*p53*+/+^ cell line and immunoprecipitation was carried out with anti-RPS20 antibody followed by western blot with anti-GFP antibody. Results in Fig. 3c clearly showed that GNL1 interacts with RPS20 (lane 2). As expected, GNL1^1–50^ interacted with RPS20 like GNL1^1–607^ (Fig. 3c, lane 3), validates the results of GST pull down assay. There was no interaction observed between GNL1^51–607^ and RPS20 (Fig. 3c, lane 4) further confirms that GNL1^1–50^ is critical for RPS20 interaction. As noted from the amino acid sequences, GNL1^1–50^ is rich in lysines and arginines and to identify the amino acids that are critical for GNL1 interaction with RPS20, GNL1^1–607_R22K23A^ was generated by exchanging the conserved Arg22 and Lys23 with alanine (Supplementary Fig. S2d). Interestingly, results from the co-immunoprecipitation assay indicate that replacement of Arg22 and Lys23 abrogated GNL1 interaction with RPS20 (Fig. 3c, lane 5). Collectively, data suggests that Arg22 and Lys23 residues within GNL1^1–50^ is critical for its interaction with RPS20. To confirm the conservation of GNL1 and RPS20 interaction, a panel of cell lines (HCT116^*p53*+/+^, AGS and HEK293T) were selected and tested. GNL1 and RPS20 complexes from cells lysates were co-immunoprecipitated with anti-RPS20 antibodies followed by western blot with anti-GNL1 (HCT116^*p53*+/+^ and AGS) or anti-GFP (HEK 293T) antibodies. Results in Fig. 3d clearly demonstrate that the existence of GNL1 and RPS20 complexes in mammalian cells (lane 2, 4 and 6). No interaction was observed with control IgG confirms the specificity of GNL1 and RPS20 interaction. Taken together, these data suggest that RPS20 is a novel interacting partner of GNL1.

### GNL1 modulates RPS20 level in mammalian cells

**Figure 4:**
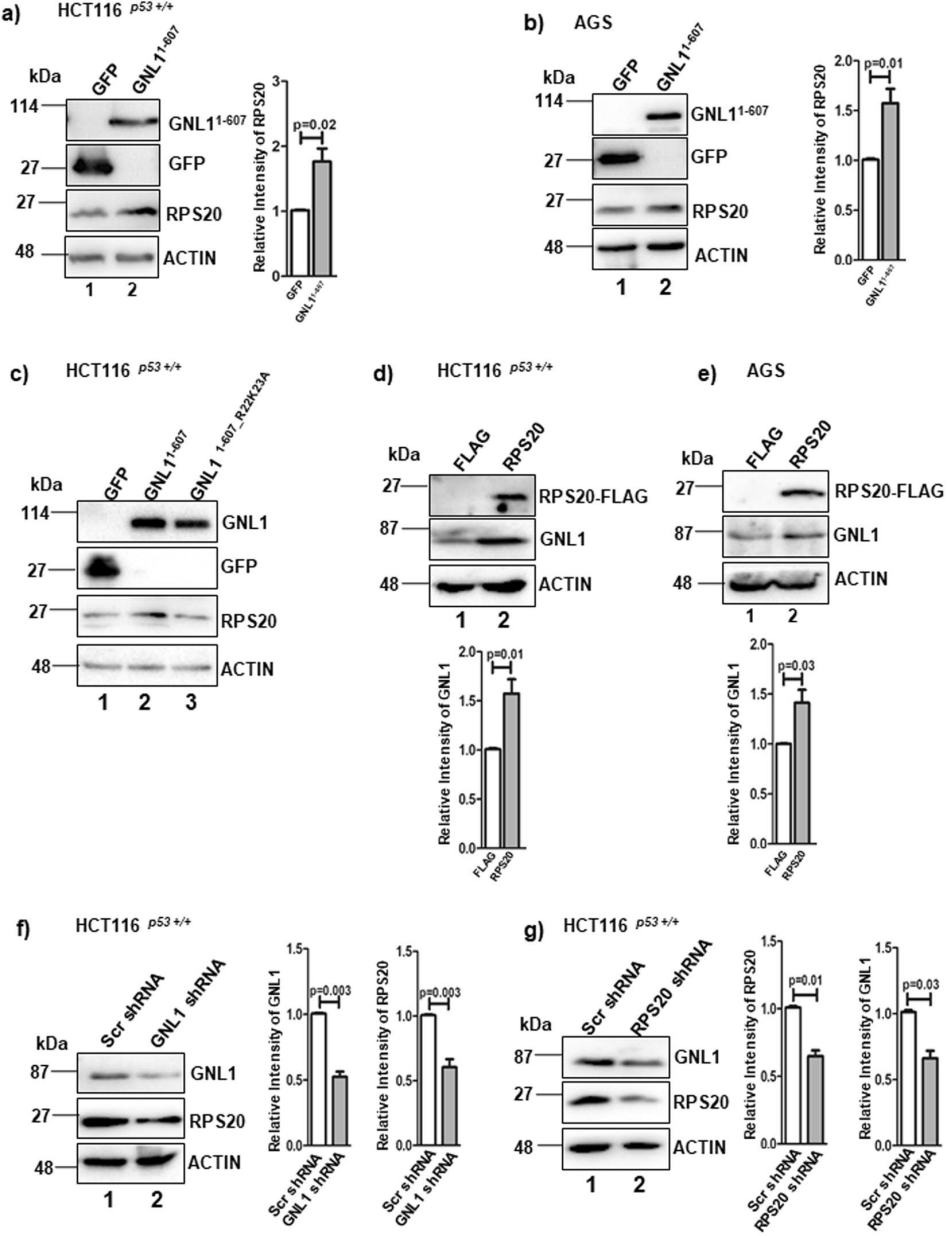
GNL1 positively modulates RPS20 expression. (**a**) HCT116^*p53*+/+^ and (**b**) AGS cells were transfected with GNL1^1–607^ or control GFP vector. After 48 hours of transfection, the levels of ectopically expressed GNL1 and endogenous RPS20 were checked by western blotting using anti-GFP and anti-RPS20 antibodies, respectively. (**c**) HCT116^*p53*+/+^ were transfected with GNL1^1–607^, GNL1^1–607_R22K23A^ or control GFP vector and the levels of all indicated proteins were determined by western blot analysis using respective antibodies. (**d**) HCT116^*p53*+/+^ and (**e**) AGS cells were transfected with RPS20-FLAG or FLAG vector. The endogenous GNL1 and RPS20-FLAG levels were determined by western blot analysis using anti-GNL1 and anti-FLAG antibodies, respectively. (**f** and **g**) HCT116^*p53*+/+^ cells were transfected with GNL1 shRNA or RPS20 shRNA or scrambled shRNA. After 72 hours of transfection, the knockdown efficiency and the expression of GNL1 and RPS20 was determined by western blot analysis using anti-GNL1 and anti-RPS20 antibodies, respectively. In (**a**–**g**) β-actin was used as loading control. The full-length blots are presented in Supplementary Fig. 10. The densitometry analyses of the corresponding western blots were carried out by normalizing the expression levels of the indicated endogenous proteins to β-actin and error bar indicates SD of three independent experiments.

To understand the functional significance of GNL1-RPS20 interaction, we first tested the endogenous RPS20 protein levels upon ectopic expression of GNL1^1–607^ in HCT116^*p53*+/+^ and AGS cell lines. Ectopic expression of GNL1^1–607^ resulted in significant increase of endogenous RPS20 protein levels in both cell lines tested (Fig. 4a and b, lane 2). Interestingly, the levels of RPS20 was not altered upon expression of the RPS20 interaction deficient mutant of GNL1 (GNL1^1–607_R22K23A^) in HCT116^*p53*+/+^ cells (Fig. 4c; lane 1 and 3). In addition, endogenous GNL1 protein level was significantly increased upon ectopic expression of RPS20-FLAG (here after referred as RPS20) in HCT116^*p53*+/+^ and AGS cell lines (Fig. 4d and e, lane 2). Interestingly, we observed the upregulation of RPS20 mRNA levels in HCT116^*p53*+/+^ cells upon GNL1^1–607^ overexpression whereas ectopic expression of RPS20 did not alter the GNL1 mRNA levels (Supplementary Fig. S3a). Furthermore, GNL1 knockdown resulted in reduction of RPS20 protein levels in both HCT116^*p53*+/+^ and AGS cell lines (Figs 4f and S3b) and the RPS20 depletion downregulated GNL1 levels in HCT116^*p53*+/+^ cells (Fig. 4g). The efficiency of GNL1 and RPS20 knockdown was checked by western blotting with respective antibodies. The above data lead to the hypothesis that RPS20 increasing GNL1 protein levels by altering its stability. Further to understand the mechanism of RPS20 mediated upregulation of GNL1, cycloheximide (CHX) chase assay was performed in HCT116^*p53*+/+^ cells to determine the stability of GNL1. Results in Supplementary Fig. S3c suggest that the ectopic expression of RPS20 increased GNL1 protein stability compared to vector transfected cells. Together, these data suggest that GNL1 modulates RPS20 at both transcriptional and translational level whereas RPS20 regulates GNL1 protein stability.

### RPS20 promotes G1/S phase progression during cell cycle

**Figure 5:**
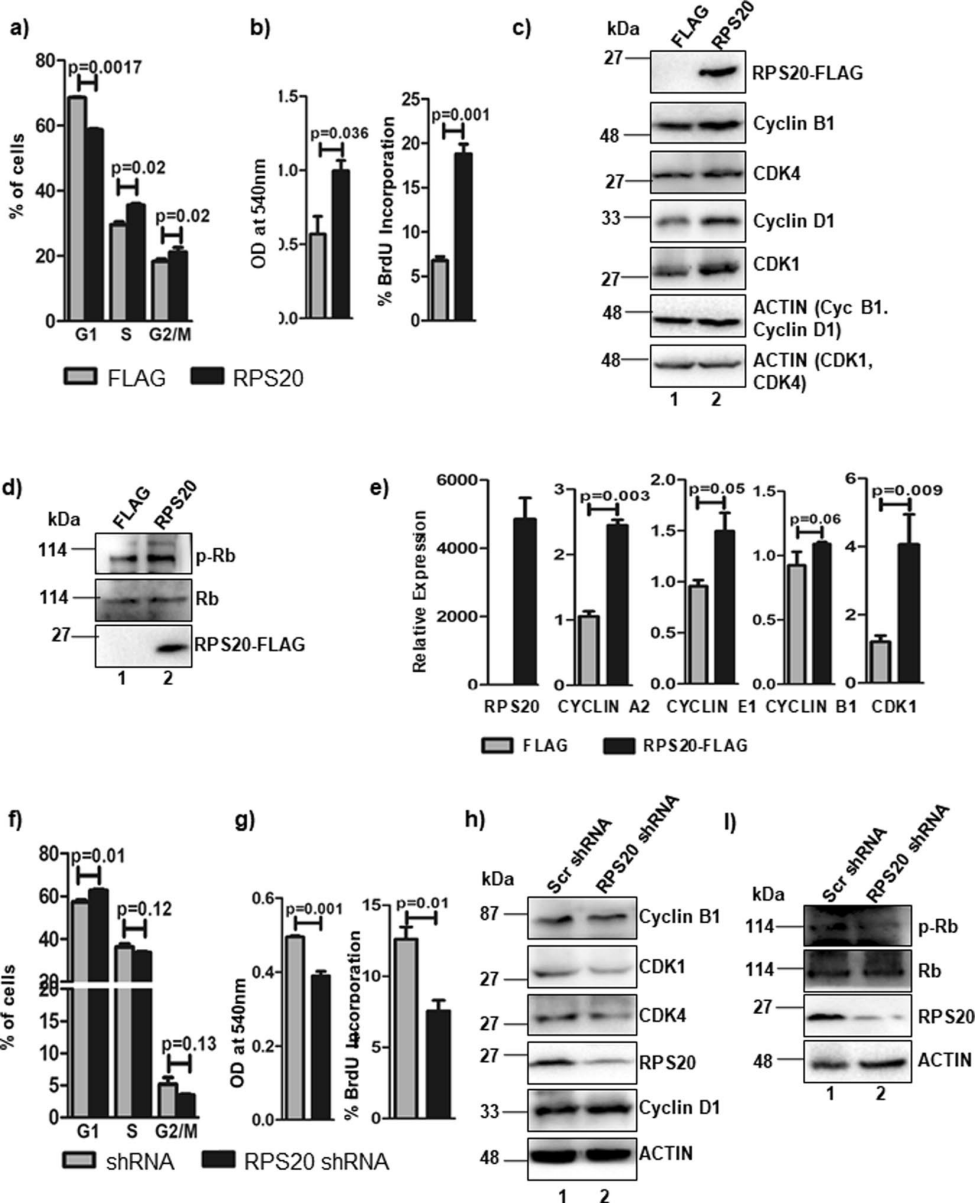
RPS20 regulates G1/S phase progression. (**a**) RPS20 was ectopically expressed in HCT116^*p53*+/+^ cells and cell cycle profile was analyzed using Flow Cytometry. (**b**) Cell proliferation was assessed by MTT and BrdU incorporation assay in HCT116^*p53*+/+^ cells expressing RPS20. (**c**) Western blot analysis was performed to analyze the levels of endogenous Cyclin B1, CDK4‚ Cyclin D1 and CDK1 upon RPS20 expression in HCT116^*p53*+/+^ cells. (**d**) Rb phosphorylation status was determined upon ectopic expression of RPS20. Anti-phospho Rb(S780) and anti-Rb antibodies were used to detect the phospho and total Rb protein levels, respectively. (**e**) RT-qPCR was carried out to analyze the expression profiles of cyclin A2, cyclin E1, cyclin B1 and CDK1 upon RPS20 overexpression. (**f**) RPS20 shRNA or scrambled shRNA was transfected in HCT116^*p53*+/+^ cells and cell cycle profile was determined using Flow Cytometry. (**g**) Cell proliferation was assessed by MTT and BrdU incorporation assay upon RPS20 knockdown in HCT116^*p53*+/+^. (**h**) Cyclin B1, CDK1, CDK4 and cyclin D1 levels were determined by western blot analysis in HCT116^*p53*+/+^ cells under RPS20 Knockdown condition. (**i**) The expression levels of phosphorylated and total Rb protein were determined by western blot analysis upon knockdown of RPS20 using anti-phospho Rb(S780) and anti-Rb antibodies respectively. β-actin was used as loading control. The full-length blots are presented in Supplementary Fig. 11.

Earlier reports suggest that RPS20 modulates cell proliferation^28,29^, but the mechanism(s) is not yet defined. To understand the physiological relevance of GNL1 and RPS20 interaction, we first tested whether RPS20 expression alters the proliferation of cycling cells. Towards this, RPS20 was transiently expressed in HCT116^*p53*+/+^ cells and the cell cycle profile was determined by flow cytometry. RPS20 significantly altered the G1/S phase of cell cycle as indicated by lesser number of cells accumulated in G1 phase with a subsequent increased accumulation of cells in S and G2/M phases (Fig. 5a). To further confirm, cell proliferation was measured by MTT and BrdU incorporation assays in RPS20 expressing HCT116^*p53*+/+^ cells. Results in Fig. 5b clearly indicate that RPS20 overexpression resulted in significant increase of cell viability and proliferation as indicated by higher BrdU labelling. To understand the mechanism of RPS20 mediated cell cycle regulation, the levels of cyclins and CDKs were determined upon RPS20 expression. Western blot analysis indicated that the levels of cyclin D1‚ cyclinB1, CDK1 and CDK4 were significantly upregulated upon ectopic expression of RPS20 (Fig. 5c, lane 2) as same as during GNL1 expression (Fig. 2). We next tested the status of Rb phosphorylation in RPS20 expressing HCT116^*p53*+/+^ cells. Western blot analysis convincingly indicated that the level of pRb^S780^ significantly increased by RPS20 without altering total Rb protein level (Fig. 5d, lane 2). This leads to the hypothesis that the observed increased pRb^S780^ levels might result in the release of E2F1 from Rb-E2F1 inhibitory complex to transactivate E2F1 target genes. Towards this, we tested the effect of RPS20 expression on mRNA levels of E2F1 target genes. Results in Fig. 5e indicate that the RPS20 significantly altered the expression of cyclin A2, cyclin E1, cyclin B1 and CDK1. Interestingly, knockdown of RPS20 did not significantly alter the cell cycle profile (Fig. 5f). To further define whether RPS20 knockdown alters cell viability/proliferation, MTT and BrdU incorporation assays were performed. Results in Fig. 5g indicate that cell viability and proliferation were significantly reduced upon RPS20 knockdown. Interestingly, RPS20 knockdown significantly decreased the levels of CDK4 and CDK1 without altering cyclin B1 and cyclin D1 expression (Fig. 5h, lane 2). Efficiency of RPS20 knockdown was determined by western blot analysis using anti-RPS20 antibodies (Fig. 5h). Result in Fig. 5i indicate that the level of pRb^S780^ was significantly decreased in RPS20 depleted cells (lane 2). Collectively, these results suggest that RPS20 promotes G1/S phase of the cell cycle by altering the status of Rb phosphorylation.

### GNL1 interaction with RPS20 is critical to promote cell proliferation

**Figure 6:**
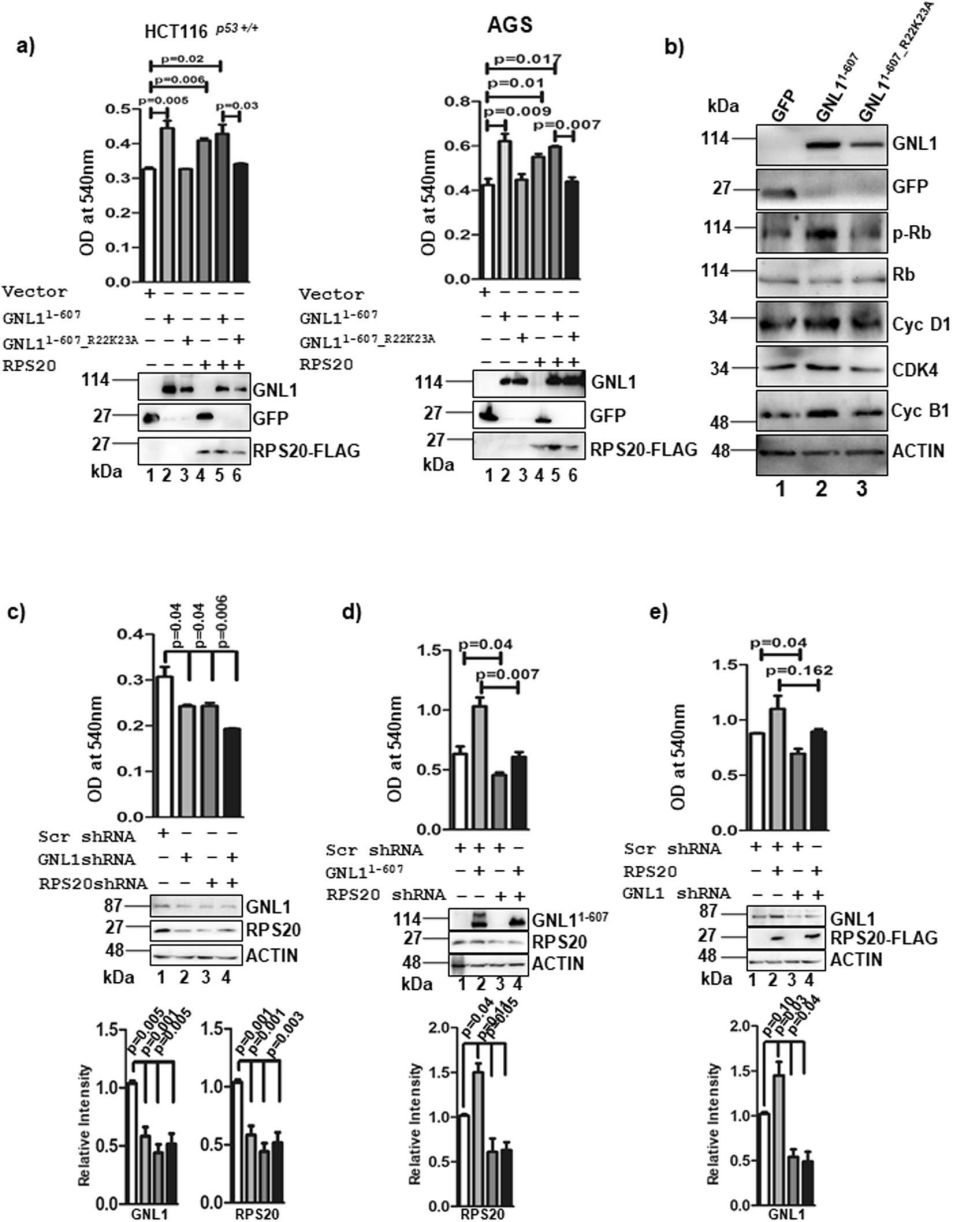
GNL1 interaction with RPS20 is critical for promoting cell growth. (**a**) MTT assay was performed to check the cell viability of HCT116^*p53*+/+^ and AGS cells transfected with GNL1^1–607^ or GNL1^1–607_R22K23A^ or RPS20-FLAG alone or in combination. (**b**) Upon ectopic expression of GNL1^1–607^ or GNL1^1–607_R22K23A^ or control vector, the levels of pRb^S780^, total Rb, cyclin D1, CDK4 and cyclin B1 were determined by western blot analysis. (**c**) MTT assay was performed to check the cell viability of HCT116^*p53*+/+^ transfected with GNL1 and RPS20 specific shRNAs alone or in combination (**d**) ectopically expressed GNL1^1–607^ or control vector under RPS20 knockdown condition (**e**) Ectopically expressed RPS20-FLAG under GNL1 depleted condition. The bar diagram depicts the densitometry analyses of the western blots by normalizing the expression levels of the indicated endogenous proteins to β-actin and error bar indicates SD of three independent experiments. The full-length blots are presented in Supplementary Fig. 12.

To understand the physiological significance of GNL1 interaction with RPS20 during cell proliferation, wild type and RPS20 interaction deficient mutant of GNL1 (GNL1^1–607_R22K23A^) were transiently expressed alone or in combination with RPS20 in HCT116^*p53*+/+^ and AGS cells and analyzed cell viability. Results from MTT assay clearly indicated that expression of GNL1^1–607^ alone or in combination with RPS20 resulted in increased viability of both HCT116^*p53*+/+^ and AGS cells (Fig. 6a). In contrast, co-expression of GNL1^1–607_R22K23A^ with RPS20 failed to promote cell viability (Fig. 6a). Expression levels of GNL1 and RPS20 was determined by western blot analysis. These results clearly indicate that the GNL1^1–607_R22K23A^ failed to promote cell growth as compared to GNL1^1–607^. To further define the mechanism, we tested the levels of Rb phosphorylation and cell cycle regulatory proteins upon expression of GNL1^1–607_R22K23A^. Results in Fig. 6b demonstrate that the levels of pRb^S780^, Cyclin D1, CDK4 and Cyclin B1 were not altered upon GNL1^1–607_R22K23A^ expression compared with GNL1^1–607^ (lane 2 and 3). These data suggest that GNL1 interaction with RPS20 is prerequisite for GNL1 to induce cell proliferation. To further understand the specificity of GNL1 and RPS20 interaction on cell proliferation, GNL1 and RPS20 were depleted alone or in combination by specific shRNAs in HCT116^*p53*+/+^ cells and determined the cell viability by MTT assay. Results in Fig. 6c suggest that knockdown of both GNL1 and RPS20 resulted in significant reduction of HCT116^*p53*+/+^ cell viability. To further define the RPS20 dependency for GNL1 function, GNL1 was overexpressed under RPS20 knockdown condition and measured cell viability. Results indicated that under RPS20 depleted condition, GNL1 was not able to promote cell survival (Fig. 6d). To further understand the role of RPS20 in GNL1 induced cell survival, the level of pRb^S780^ was checked with cells expressing GNL1 with or without RPS20 knockdown. Results indicate that ectopic expression of GNL1^1–607^ under RPS20 knockdown condition failed to upregulate pRb^S780^ level (Supplementary Fig. S4a). Together, these results strongly suggest the critical requirement of RPS20 in GNL1 induced cell proliferation. Similarly, ectopic expression of RPS20 under GNL1 knockdown condition failed to promote cell viability (Fig. 6e). Scrambled shRNA was used as negative controls. Expression and knockdown efficiencies of GNL1 and RPS20 were determined by western blot analysis with respective antibodies. Taken together, these data provided evidence that GNL1 interaction with RPS20 is critical to promote cell proliferation during cell division cycle.

### GNL1 promotes cell survival in co-operation with RPS20

**Figure 7:**
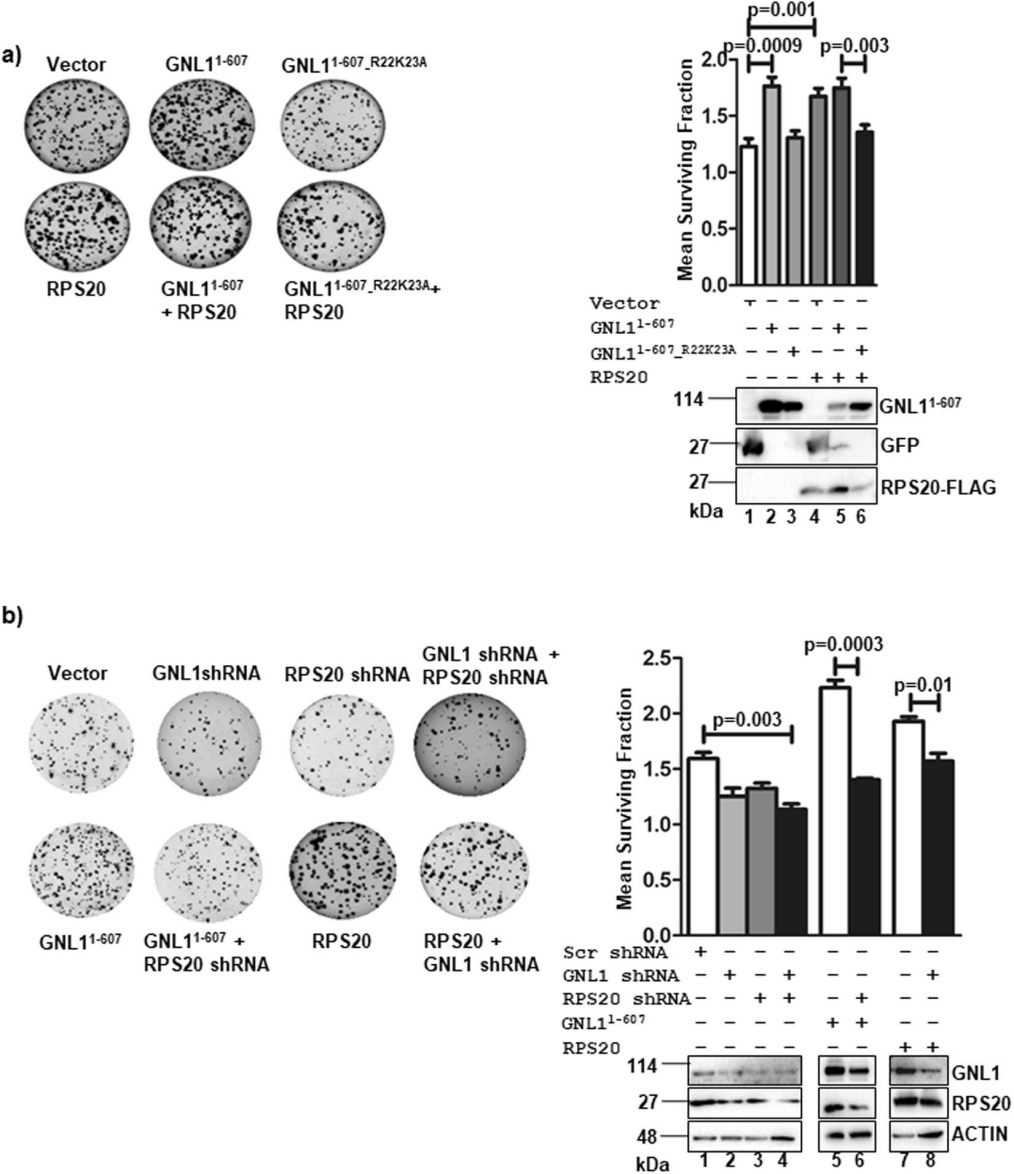
GNL1 interaction with RPS20 is critical for cell survival. (**a**) Colony forming assay was carried out in HCT116^*p53*+/+^ cells by expressing GNL1^1–607^ or GNL1^1–607_R22K23A^ or RPS20-FLAG alone or in combination. After 48 hours of transfection, GFP positive cells were sorted and selected in cell growth medium containing G418 for 14 days. (**b**) Colony forming ability of HCT116^*p53*+/+^ cells were determined by depleting GNL1 and/or RPS20 using specific shRNAs with or without GNL1^1–607^ or RPS20 expression. The mean surviving fraction of HCT116^*p53*+/+^ cells expressing indicated proteins was calculated and plotted as described in Materials and Methods. Expression of all indicated proteins were determined by western blot analysis using respective antibodies. The full-length blots are given in Supplementary Fig. 13.

To understand the physiological importance of GNL1 and RPS20 interaction during tumorigenesis, the colony formation ability of HCT116^*p53*+/+^ or AGS cells were checked upon ectopic expression or knockdown of GNL1 with or without RPS20. Toward this, HCT116^*p53*+/+^ or AGS cells were transfected with GNL1^1–607^ or GNL1^1–607_R22K23A^ or RPS20 alone or in combination and determined the colony forming ability as described in Materials and Methods. Results indicate that the number of colonies were significantly increased upon the expression of GNL1^1–607^ or RPS20 alone or in combination (Figs 7a and S4b). In contrast, the colony numbers were significantly reduced when HCT116^*p53*+/+^ or AGS cells were co-expressed with GNL1^1–607_R22K23A^ and RPS20 (Figs 7a and S4b). The mean surviving fraction of HCT116^*p53*+/+^ or AGS cells were calculated as described in Materials and Methods. Together, these data suggest that GNL1 interaction with RPS20 is critical to promote cell proliferation/survival. To further define the role played by GNL1/RPS20 complex on cell proliferation, GNL1 and RPS20 was depleted individually or in combination with specific shRNAs in HCT116^*p53*+/+^ cells and the colony forming ability was measured. Results in Fig. 7b indicate that the number of colonies and sizes were drastically reduced when GNL1 depleted together RPS20. To further confirm the RPS20 dependency on GNL1 function during cell proliferation, the colony formation ability of HCT116^*p53*+/+^ cells was measured with GNL1 expression under RPS20 knockdown or RPS20 expression under GNL1 knockdown conditions. Interestingly, GNL1 expression under RPS20 depletion condition failed to promote the colony forming ability of HCT116^*p53*+/+^ cells as indicated by decreased number and sizes of colonies (Fig. 7b). Similar trend was observed when RPS20 was expressed under GNL1 knockdown condition (Fig. 7b). Expression and depletion levels of GNL1 and RPS20 were determined by western blot analysis using respective antibodies. The bar diagram represented the mean surviving fraction of HCT116^*p53*+/+^ cells. Collectively, these data suggest that GNL1 promotes cell survival in RPS20 dependent manner.

### Positive co-relation of GNL1 and RPS20 expression in human cancers

**Figure 8:**
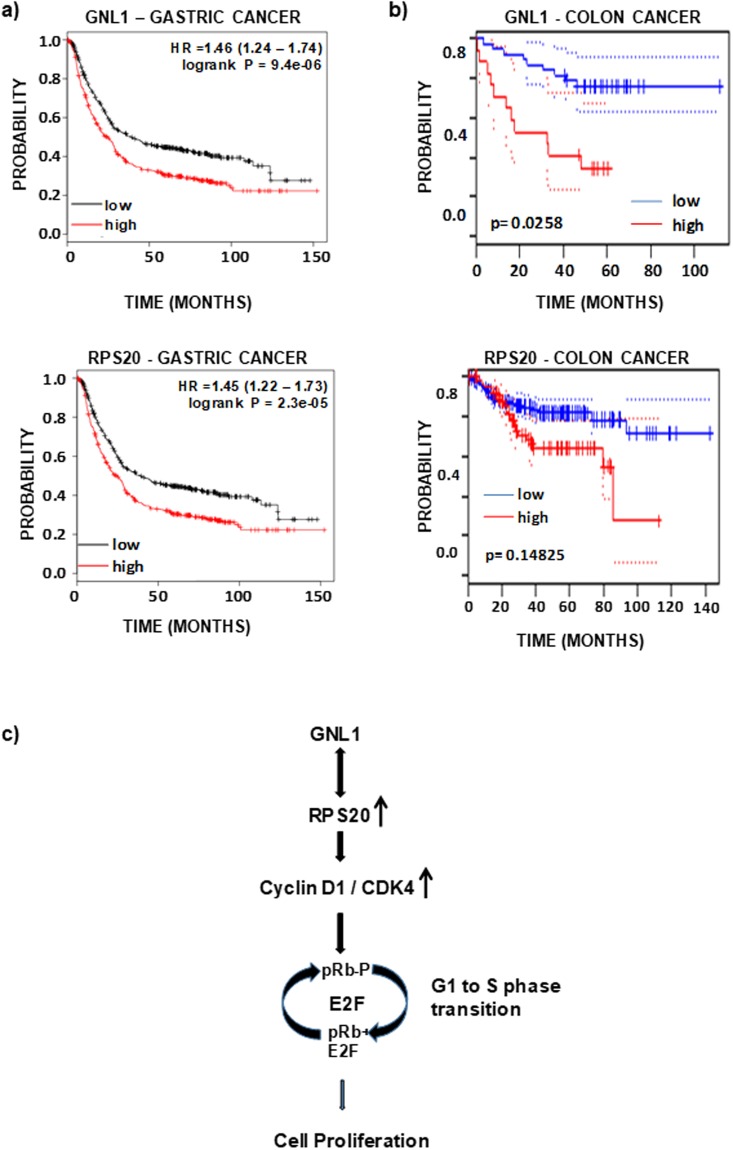
Expression of GNL1 and RPS20 inversely correlated with gastric and colon cancer patient survival. (**a**) Kaplan Meier (KM) plot of overall survival in gastric cancer patients (n = 876) based on the expression status of GNL1 and RPS20. (**b**) Kaplan-Meier curves of overall survival of colorectal cancer patients inversely correlated with GNL1 (n = 79) and RPS20 (n = 226) expression status from Prognoscan database. (**c**) Proposed model of GNL1 and RPS20 function during cell proliferation. Results from the present investigation clearly illustrates that (**a**) GNL1 interacts with RPS20, (**b**) Interaction between GNL1 and RPS20 is critical to regulate cell proliferation and (**c**) GNL1 and RPS20 modulates G1/S and G2/M checkpoint of cell cycle by altering the status of Rb phosphorylation to promote cell proliferation.

The analysis of *in vivo* mRNA expression patterns of GNL1 and RPS20 in colon and gastric cancers from BioXpress database^22^ indicates a positive correlation (Supplementary Fig. S5a and b). This data together with our results support notion that the interplay between GNL1 and RPS20 is critical to promote cell survival and proliferation. We next evaluated the prognostic effect of GNL1 and RPS20 gene expression in gastric cancer tissues by Kaplan-Meier Plotter analysis from the publicly available database (www.kmplot.com)^30^. Kaplan-Meier analysis showed that high mRNA expression of GNL1 and RPS20 was strongly associated with decreased overall survival of gastric cancer patients (Fig. 8a). Also, assessed the effect of expression status of GNL1 and RPS20 in overall survival of colorectal cancer patients from the tissue data available in Prognoscan database (Fig. 8b)^31^. The results from this analysis indicate the existence of negative correlation between expression of GNL1 and RPS20 and patient survival. Collectively, these results provide evidence that the interplay between GNL1 and RPS20 play an important role during tumorigenesis.

## Discussion

In the present study, we have attempted to gain deeper understanding of GNL1 function during cell proliferation. Using yeast-two hybrid assay, RPS20 was identified as a novel functional interacting partner of GNL1 and showed that interaction between these proteins is critical to promote cell proliferation and survival. The inability of RPS20 interaction deficient mutant of GNL1 (GNL1^1–607_R22K23A^) to promote cell proliferation supports the notion that RPS20 plays critical role in GNL1 function. Expression of GNL1 and RPS20 induced the colony forming ability of colon and gastric cancer cell lines provided evidence for the role played by GNL1 and RPS20 during cancer progression. In addition, analysis of gene expression databases suggested that the expression of GNL1 and RPS20 in primary colon and gastric cancers inversely correlated with patient survival further strengthen their critical role in tumorigenesis.

GNL1 was initially identified as nucleolar G-domain containing protein belongs to YawG/YIqF subfamily of GTPases^27^. The previous report from our laboratory showed that GNL1 harbors a novel arginine/lysine rich nucleolar localization signal and localizes to different subcellular compartments in cell cycle dependent manner^10^. Unlike GNL3L, GNL1 shuttles between nucleus and cytoplasm via CRM1 independent pathway and GTP binding is critical for GNL1 shuttling between nucleus and nucleolus^10^. Interestingly, gene expression analysis of cancer tissue data from BioXpress database showed that GNL1 is overexpressed in majority of cancers, supporting the notion that there is positive correlation of GNL1 expression with cell proliferation^22^ but the pathophysiological relevance of its upregulation during tumorigenesis remains poorly understood.

Towards understanding the function of GNL1, using yeast two hybrid system, Ribosomal Protein S20 (RPS20) was identified as a novel functional interacting partner of GNL1. RPS20 is evolutionarily conserved across the metazoa from yeast to human. In homo-sapiens, *rps20* expresses two transcript variants – isoforms 1 and 2^32^. RPS20 isoform 1 contains 142 amino acids while isoform 2 encodes 119 amino acids. Previous report suggests that RPS20 play an important role in ribosomal RNA processing^33^ and predominantly localized in cytoplasm^34^ which is distinct from other ribosomal proteins that are commonly found in nucleolus^35–40^. Recent studies provided clear evidence that several ribosomal proteins play a critical role in the regulation of ‘non-ribosomal function’ like modulating p53-MDM2 pathways to promote cell proliferation during tumorigenesis^41–52^. RPS20 interacts with MDM2^52^ and stabilizes p53 by inhibiting MDM2 ubiquitin ligase activity^29^.

Results from the current investigation clearly indicates that ectopic expression of GNL1 upregulates RPS20 expression and promotes cell proliferation. GNL1 function is severely impaired upon RPS20 knockdown which shows the critical role played by RPS20 in GNL1 function. Interestingly, higher expression of RPS20 is correlated with poor survival of medulloblastoma patients^53^ and considered as a novel predictor of poor prognosis of Glioblastoma^54^. In addition, RPS20 was found to be upregulated in human colon carcinoma cell lines^55^. Further, the ectopic expression of RPS20 upregulates GNL1 by protein stabilization. Analysis of *in vivo* expression pattern of GNL1 and RPS20 in colon and gastric cancers from BioXpress database indicates positive co-relation, supporting the notion that GNL1-RPS20 co-operation may be important for cell growth during tumorigenesis.

Interestingly, GNL1 and RPS20 upregulate the levels of cyclin D1 and CDK4, thereby promoting faster G1/S cell cycle phase progression. The G1/S cell cycle phase transition is controlled by Rb by forming an inhibitory complex with E2F1 and prevents E2F1 dependent transcription. Results from the current study clearly demonstrate that the ectopic expression of GNL1 and RPS20 modulates the mRNA level of cyclin A2, Cyclin E1, Cyclin B1 and CDK1 to promote faster cell cycle progression. The deregulation of G1/S restriction point leads to uncontrolled cell proliferation and contributes to tumorigenesis. Rb is phosphorylated by cyclinD-cdk4/6 complex during ‘G1/S’ phase of the cell cycles^56^. The proposed functional model (Fig. 8c) based on the data from the present study suggests that GNL1 and RPS20 upregulates the level of cyclin D1 and CDK4, thereby increasing cyclin D1/CDK4 complex formation and resulted in hyperphosphorylation of Rb on serine 780, which is critical to release E2F1 from Rb-E2F1 inhibitory complex to transactivate its target to promote cell division cycle.

Collectively, the present study demonstrates that RPS20 is a novel interacting partner of GNL1. GNL1 induces cell proliferation by modulating RPS20 level. The observed high expression levels of GNL1 and RPS20 in various cancer tissues together with our results from the current study suggests the possibility that the co-operation of GNL1 and RPS20 may favor faster cell proliferation during cancer progression and may be an ideal target for cancer therapeutic intervention.

## Material and Methods

### Plasmid Construction

GFP tagged GNL1 and its deletions were generated as described previously^10^. Site-specific mutant GNL1^1–607_R22K23A^ was generated by Quick-change mutagenesis (Stratagene, USA) using appropriate primers (Supplementary Table S1). RPS20 isoform 2 open reading frame was amplified from HeLa cDNA library using appropriate primers (Supplementary Table S1) and cloned as GST and FLAG fusion protein in pCDNA3. DNA sequencing was performed to validate the integrity of all the plasmids.

### Antibodies

Anti-FLAG and anti-beta actin (Sigma-Aldrich, USA); anti-GNL1, anti-RPS20, anti-CDK1 and anti-MDM2 (Abcam, UK); anti-GFP, anti-Cyclin B1 and anti-p53 (Santa Cruz, USA); anti-p21, anti-Cyclin D1, anti-CDK4, anti-pRbSer-780, anti-Rb and anti-E2F1 (Cell Signaling Technology Inc., USA) antibodies were used in western blot analysis for checking protein expression.

### Cell Culture, Transfection and Western blot analysis

HCT116^*p53*+/+^, HCT116^*p53*−/−^, AGS and HEK293T cells were cultured in Dulbecco’s Modified Eagle’s Medium (DMEM) (Thermo Fisher Scientific Inc., USA) with 10% Fetal Bovine Serum (FBS) and 1% antibiotics-antimycotics (Thermo Fisher Scientific Inc., USA). Cells were grown to 60% confluency and transfected with polyethyleneimine (PEI) or lipofectamine 2000 (Thermo Fisher Scientific Inc., USA) according to manufacturer’s protocol. The western blot analysis with appropriate antibodies were performed as previously described^10^.

### Yeast Two Hybrid Assay

GNL1 full length was amplified using appropriate primers and cloned into Nde I and Sal I sites of pGBKT7 vector as fusion with GAL4 DNA-DB/c-MYC and used as a bait. Human leukocyte cDNA library was cloned into EcoRI and XhoI sites of pAct2 vector as fusion with GAL4-AD/HA and used as prey. Bait plasmid was first transformed into AH109 strain of *Saccharomyces cerevisiae* (Clontech, USA) and selected GNL1 positive yeast clones and then transformed with prey plasmid. Positive clones were selected on synthetic dropout (SD) medium in the absence of adenine, histidine, leucine and tryptophan. Colonies with bait-prey interaction activate the expression of α-galactosidase. The very high stringency selection medium has X-α-Gal, which can be cleaved by secreted α-galactosidase and forms insoluble blue product.

### RNA interference

GNL1 shRNA (TRCN0000189397, Sigma-Aldrich, USA), RPS20 shRNA (TRCN0000117625, Sigma-Aldrich, USA) were used for knockdown of GNL1 and RPS20 expression in HCT116^*p53*+/+^ and AGS cells. The universal control shRNA (SHC016, Sigma-Aldrich, USA) was used as a negative control in all the knockdown studies.

### Expression of RPS20 and its variants as GST fusion proteins and GST pull down assay

Full length and variants of RPS20 were transformed into *E*. *coli* BL21DE3 cells and grown overnight at 37 °C. Single colony containing GST-RPS20 was inoculated into LB medium and the protein expression was induced with 1 mM IPTG at 18 °C for 12–14 hours. The GST pull down assay was carried out as described elsewhere^10^.

### Co-Immunoprecipitation

Equal amounts of HCT116^*p53*+/+^ and AGS cell lysates were incubated with anti-RPS20 antibodies for 6 hours at 4 °C and the antibody-protein complexes were eluted and resolved on SDS-12%PAGE followed by western blotting with anti-GNL1 antibody. HEK293T cells were transfected with GNL1^1–607^ or GFP as described above. The expression levels of transfected proteins were normalized and co-immunoprecipitation was performed using anti-RPS20 antibodies followed by western blotting with anti-GFP antibody.

### Cycloheximide (CHX) chase assay

HCT116^*p53*+/+^ cells were transfected with RPS20-FLAG as described above. The cells were treated with Cycloheximide (100 μg/ml) after 48 hours of transfection and were harvested at various time intervals. The cells were lysed and equal amount of protein was loaded on SDS-12%PAGE and the resolved proteins were transferred to PVDF membrane followed by western blotting using anti-GNL1, anti-FLAG and anti-beta actin antibodies.

### Cell Cycle Analysis

HCT116^*p53*+/+^ cells were transfected with GNL1^1–607^, GNL1-shRNA, RPS20-FLAG, RPS20-shRNA, control vectors alone or in combination. After 48 hours of transfection, cell cycle analysis was carried out as detailed elsewhere^6^.

### MTT assay

HCT116^*p53*+/+^ or AGS cells were transfected with GNL1^1–607^, GNL1^1–607_R22K23A^, RPS20-FLAG, GNL1 shRNA, RPS20 shRNA alone or in combination. MTT assay was perfomed as described previously^57^.

### BrdU Incorporation assay

HCT116^*p53*+/+^ cells were transfected with GNL1^1–607^, RPS20-FLAG, GNL1 shRNA, RPS20 shRNA alone or in combination. BrdU (10 µM) was added to the transfected cells and incubated for 3 hours at 37 °C. Cells were fixed, permeablized and stained with anti-BrdU antibodies conjugated with APC. The BrdU positive cell populations were analyzed using FACS Canto II (BD Biosciences, USA).

### Colony Formation Assay

HCT116^*p53*+/+^ and AGS cells were transfected with GNL1^1–607^, GNL1^1–607_R22K23A^, RPS20-FLAG, GNL1 shRNA, RPS20 shRNA alone or in combination. After 48 hours of transfection, cells were trypsinized and GFP positive cells were sorted using MoFlo Astrios EQ Cell Sorter (Beckman Coulter, USA) in 6 wells plates. After 14 days of G418 selection, the colonies were fixed with 10% neutral buffered formaldehyde (5 ml of formaldehyde + 0.2 g sodium dihydrogen phosphate + 0.325 g disodium hydrogen phosphate) and stained with 0.01% crystal violet. The colony number were counted using ImageJ software. The surviving fraction of cells was determined by dividing the plating efficiency of cells transfected with GNL1^1–607^, GNL1^1–607_R22K23A^, RPS20-FLAG, GNL1 shRNA, RPS20 shRNA alone or in combination by plating efficiency of control cells. The mean surviving fraction was calculated by averaging the surviving fraction of triplicates and values were plotted as bar diagram using graph pad prism 5.0 software.

### qPCR analysis

Total RNA from HCT116^*p53*+/+^ cells transfected with different expression plasmids was isolated using TRIzol reagent (TaKaRa, Japan). qPCR analysis was performed as described previously^57^. The expression levels of various genes relative to beta-actin were analyzed according to the manufacturer’s directions. The primers used for the qPCR analyzes are given in Supplementary Table 1.

### Gene Expression Analysis from BioXpress Database

Gene expression levels are mapped to genes using RNA-seq of 920 pairs of tumor and normal tissue samples, which are available in the BioXpress database^22^ obtained from the TCGA database for the following cancers Esophageal carcinoma [ESCA], Cervical squamous cell carcinoma [CESC], Uterine corpus endometrial carcinoma [UCEC], Liver hepatocellular carcinoma [LIHC], Kidney renal papillary cell carcinoma [KIRP], Kidney renal clear cell carcinoma [KIRC], Colon adenocarcinoma [COAD], Rectum adenocarcinoma [READ], Pancreatic adenocarcinoma [PAAD], Thyroid carcinoma [THCA], Breast invasive carcinoma [BRCA], Lung squamous cell carcinoma [LUSC], Lung adenocarcinoma [LUAD], Head and neck squamous cell carcinoma [HNSC], Bladder Urothelial Carcinoma [BLCA], Stomach adenocarcinoma [STAD], Prostate adenocarcinoma [PRAD] and Sarcoma [SARC].

### Statistical analysis

Statistical analysis was carried out using Graph Pad Prism 5.0 software. The experiments were repeated thrice with three biological replicates. Error bars represent mean ± S.D from a representative experiment with biological triplicates. The statistical significance (p values) were obtained by performing student’s ‘t’ test (unpaired). Image J software was used for densitometry analysis of western blots. The relationship between GNL1 and RPS20 expression was assessed using Pearson correlation test performed on the data retrieved from BioXpress database. Kaplan-Meier analysis was employed to evaluate the distribution of overall survival of colon and gastric cancer patients. The statistical significance for Km plots was shown from the log rank P-value.

## Electronic supplementary material

Supplementary Table 1

Supplementary figures
