# Supplementary material for: Interplay between human nucleolar GNL1 and RPS20 is critical to modulate cell proliferation
Source: Sci Rep. 2018 Jul 30;8:11421. doi: 10.1038/s41598-018-29802-y (PMC6065441; doi:10.1038/s41598-018-29802-y)
Supplement: Supplementary file 1 — Supplementary Table 1 [file 41598_2018_29802_MOESM1_ESM.pdf]

Interplay between human nucleolar GNL1 and RPS20 is critical to modulate cell proliferation

Rehna Krishnan, Neelima Boddapati and Sundarasamy Mahalingam*

Laboratory of Molecular Virology and Cell Biology, Department of Biotechnology,

Bhupat and Jyoti Mehta School of Biosciences, Indian Institute of Technology-Madras, Chennai 600 036, India.

*Address for Correspondence:

Sundarasamy Mahalingam, Laboratory of Molecular Virology and Cell Biology, Room No: 403, Department of Biotechnology, Bhupat and Jyoti Mehta School of Biosciences, Indian Institute of Technology-Madras, Chennai 600 036, India.

Tel: (+91-44)-22574130; Fax: (+91-44)-22574102; E-mail: mahalingam@iitm.ac.in

Supplementary Table 1: Primers used for cloning and qPCR analysis

**Primer used for cloning**	**Sequence (5'-3')**
ADLD Fwd	CTA TTC GAT GAT GAA GAT ACC CCA CCA AAC CC
ADLD Rev	GTG AAC TTG CGG GGT TTT TCA GTA TCT ACG AT
pACT2 Fwd	GGC CAA GAT TGA AAC TTA GAG GAG TAT AG
pACT2 Rev	CCA CTG TCA CCT GGT TGG ACG GAC CAA AC
RPS20 Fwd BamH1	TGC GAC GGA TCC ATG GCT TTT AAGGAT ACC GGA AAA ACA CCC
RPS20 Rev Xho1	GCA TGA CTC GAG TTA AGC ATC TGC AAT GGT GAC TTC CAC CTC
RPS20 100 Rev Xho1	GCA TGA CTC GAG TTA CTG CTT AAC AAT CTC AGA AGG ACT GTG
RPS20 80 Rev Xho1	GCA TGA CTC GAG TTA GAA ACG ATC CCA CGT CTT AGA ACC TTC
RPS20 60 Rev Xho1	GCA TGA CTC GAG TTA AGT CTT GGT AGG CAT TCG AAC TGG TCC
RPS20 41 Fwd BamH1	TGC GAC GGA TCC ATG AGA GGC GCA AAA GAA AAG AAT CTC
**Primers used for qPCR**	
GNL1 Fwd	CCATTGATCAGCGAGGACTT
GNL1 Rev	AGCAGCAGACTGATTCAGCA
RPS20 Fwd	GGAAACGATCCCACGTCTTA
RPS20 Rev	TGGAAAAGGTGTGTGCTGAC
Cyclin A2 Fwd	CACTCACTGGCTTTTCATCTTC
Cyclin A2 Rev	ACCTGGACCCAGAAAACCAT
Cyclin E1 Fwd	CCCGGTCATCATCTTCTTTG
Cyclin E1 Rev	AGAAATGGCCAAAATCGACA
Cyclin B1 Fwd	CCAAAGGCCACTAGGCCT
Cyclin B1 Rev	GGGGCGGGGCCACAG
CDK1 Fwd	TTTCATGGCTACCACTTGACC
CDK1 Rev	TAAGCCGGGATCTACCATACC
Beta-actin Fwd	ATGAAGTGTGACGTGGACAT
Beta-actin Rev	GGCCAGGTACTGATGGTC
