# Supplementary material for: Interplay between human nucleolar GNL1 and RPS20 is critical to modulate cell proliferation
Source: Sci Rep. 2018 Jul 30;8:11421. doi: 10.1038/s41598-018-29802-y (PMC6065441; doi:10.1038/s41598-018-29802-y)
Supplement: Supplementary file 2 — Supplementary figures [file 41598_2018_29802_MOESM2_ESM.pdf]

Interplay between human nucleolar GNL1 and RPS20 is critical to modulate cell proliferation

Rehna Krishnan, Neelima Boddapati and Sundarasamy Mahalingam*

Laboratory of Molecular Virology and Cell Biology, Department of Biotechnology, Bhupat and Jyoti Mehta School of Biosciences, Indian Institute of Technology-Madras, Chennai 600 036, India.

*Address for Correspondence:

Sundarasamy Mahalingam, Laboratory of Molecular Virology and Cell Biology, Room No: 403, Department of Biotechnology, Bhupat and Jyoti Mehta School of Biosciences, Indian Institute of Technology-Madras, Chennai 600 036, India.
Tel: (+91-44)-22574130; Fax: (+91-44)-22574102; E-mail: mahalingam@iitm.ac.in

Supplementary Figure 1

a)

**Screening**	**A**	**B**
**No. of colonies obtained on**<br>**SD –His/-Leu/-Trp (Medium Stringency)**<br>**SD –Ade/–His/-Leu/-Trp (High Stringency)**<br>**SD X-α-Gal/–Ade/–His/-Leu/-Trp (Very High Stringency)**	 **398**<br>**33**<br>**24**	 **230**<br>**74**<br>**41**
**Total no of plasmids isolated from yeast**	**24**	**38**
**No of transformants obtained in E. coli MC1061**	**19**	**30**
**No. of plasmids isolated from E. coli MC1061**	**19 x 3**	**30 x 3**

b)

128 atggcttttaaggataccggaaaaacacccgtggagccggaggtg
1 M A F K D T G K T P V E P E V
173 gcaattcaccgaattcgaatcaccctaacaagccgcaacgtaaaa
16 A I H R I R I T L T S R N V K
218 tccttggaaaaggtgtgtgctgacttgata*agaggcgcaaaagaa*
31 S L E K V C A D L I R G A K E
263 *aagaatctcaaagtgaaaggaccagttcgaatgcctaccaagact*
46 K N L K V K G P V R M P T K T
308 *ttgagaatcactacaagaaaaactccttgtggtgaaggttctaag*
61 L R I T T R K T P C G E G S K
353 *acgtgggatcgtttccagatgagaattcacaagcgactcattgac*
76 T W D R F Q M R I H K R L I D
398 *ttgcacagtccttctgagattgttaagcagattacttccatcagt*
91 L H S P S E I V K Q I T S I S
443 *attgagccaggagttgaggtggaagtcaccattgcagatgcttaa*487
106 I E P G V E V E V T I A D A * 119

Supplementary figure 1: (a) Yeast two hybrid screening conditions. (b) Full length sequence of A14/1 (RPS20) clone. Sequences underlined with italics represent the fragment identified from yeast two hybrid screen.

Supplementary Figure 2

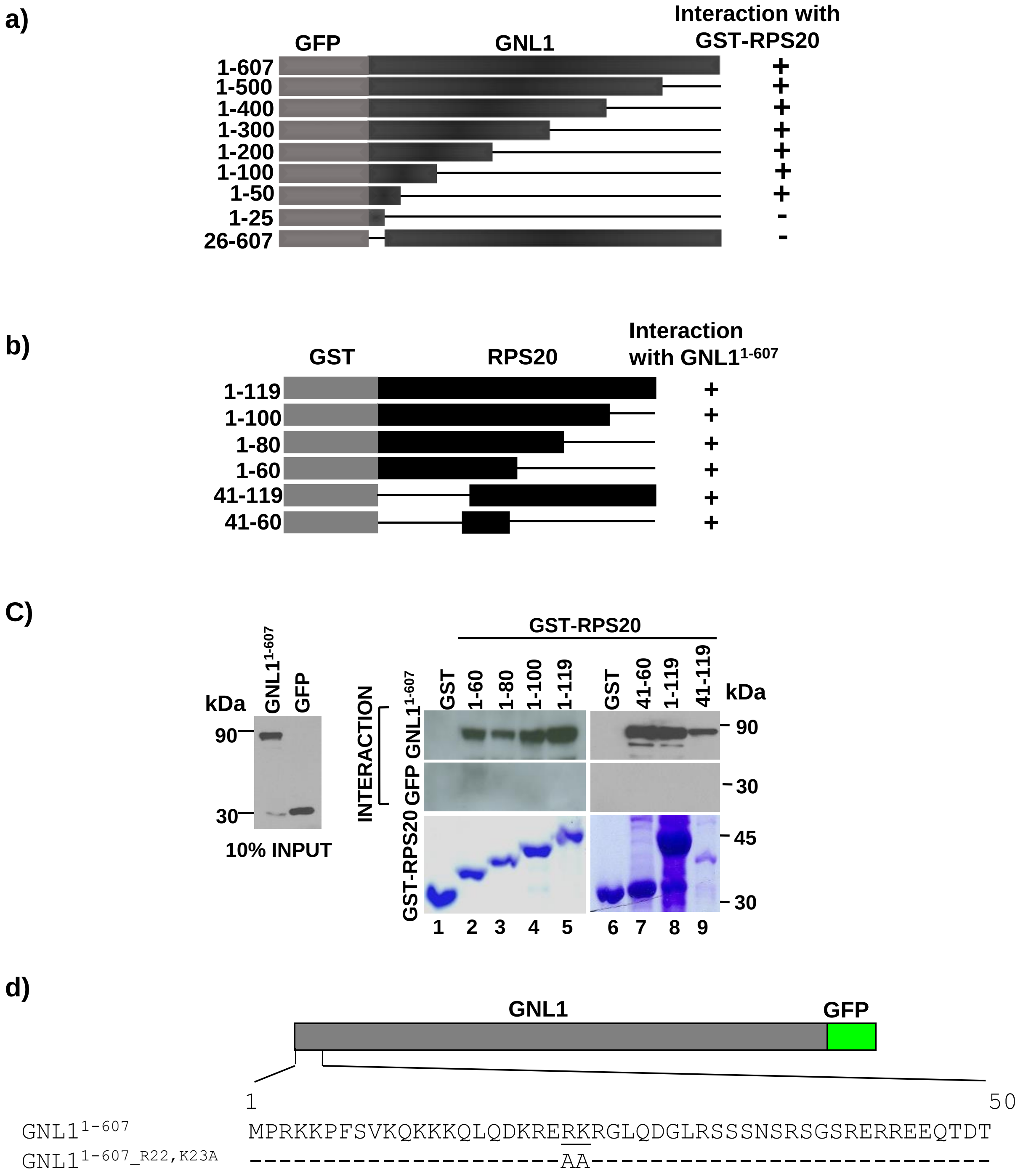

Supplementary figure 2: (a) Schematic representation of wild type and deletion mutants of GNL1 and **(b)** Wild type and deletion mutants of RPS20. **(c)** Minimal domain required for interaction with GNL1 resided between amino acids 41 to 60 of RPS20. HEK293T cell lysates containing GNL1$^{1-607}$ or GFP was added to Glutathione Sepharose beads bound with full length or variants of RPS20 fused with GST. Bound protein complexes were eluted and checked by western blot analysis using anti-GFP antibody. Coomassie blue staining was used to check the expression of GST-RPS20 and GST. **(d)** Schematic diagram indicating the conserved Arginine and Lysine residues are mutated with Alanine in GNL1$^{1-607}$ as described in Materials and Methods.

Supplementary Figure 3

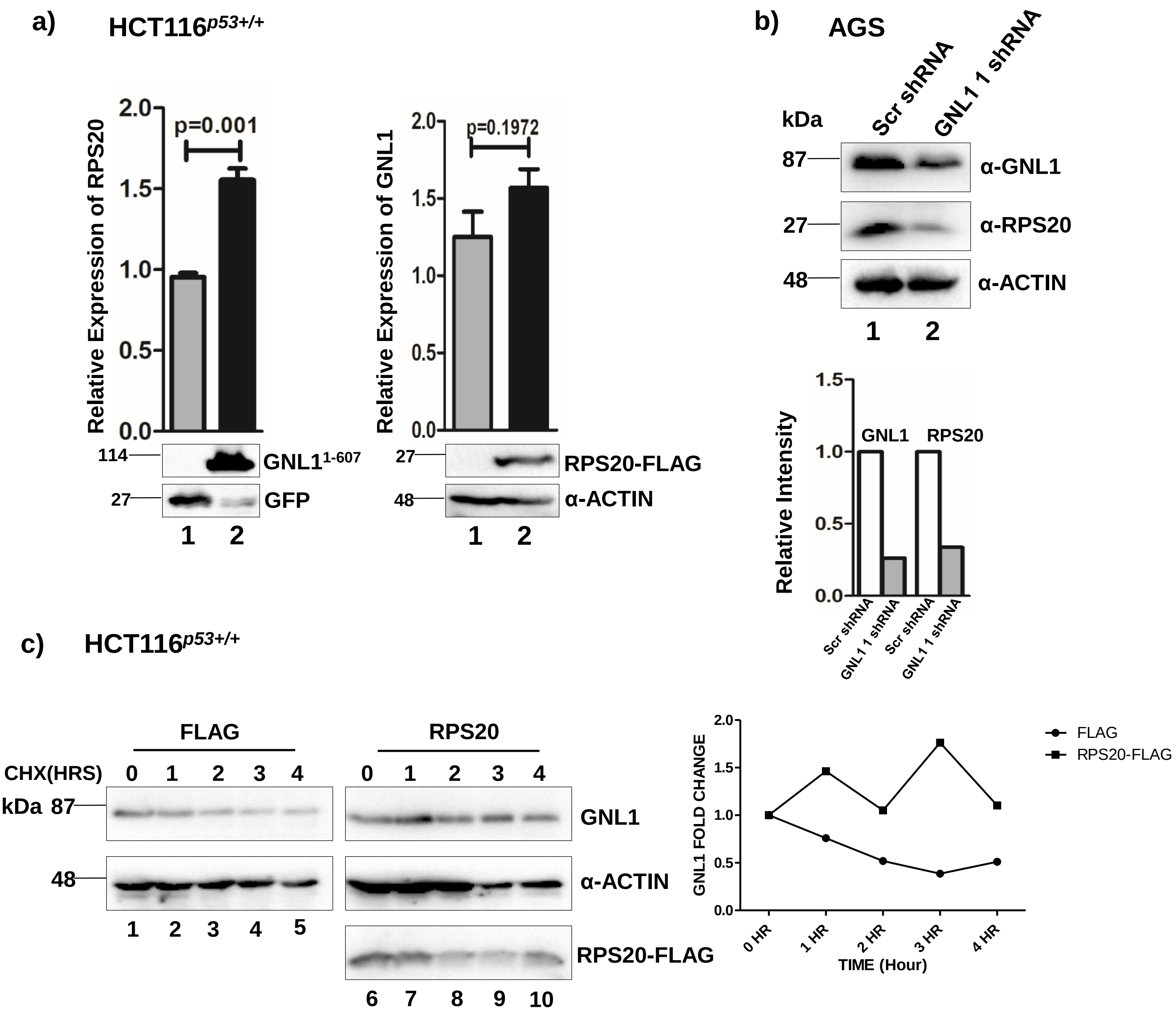

Supplementary figure 3: (a) HCT116$^{p53+/+}$ cells were transfected with GNL1$^{1\text{-}607}$, RPS20-FLAG or corresponding control vectors. After 48 hours of transfection, the total RNA was isolated and reverse transcribed. qPCR analysis was carried out using specific primers to assess the level of endogenous RPS20 or GNL1. Expression of GNL1$^{1\text{-}607}$ and RPS20-FLAG was checked by Western blot analysis using anti-GFP and anti-FLAG antibody respectively. **(b)** AGS cells were transfected with GNL1 shRNA or scrambled shRNA. After 72 hours of transfection, western blot analysis was carried out to check the endogenous level of RPS20 using anti-RPS20 antibody. The knockdown efficiency was determined by western blot analysis using anti-GNL1 antibody. The graph represents the densitometry analyses of the Western blots by normalizing the expression levels of endogenous proteins to β-actin. **(c)** HCT116$^{p53+/+}$ cells transfected with RPS20-FLAG or the control vector were treated with cycloheximide (CHX) for indicated time periods. The expression of endogenous GNL1 was determined by western blot using anti-GNL1 antibody. Beta-actin was used as loading control. The densitometry analysis of the western blot indicating the level of endogenous GNL1 at different time points. The full length blots are presented in Supplementary figure 14.

Supplementary Figure 4

a)

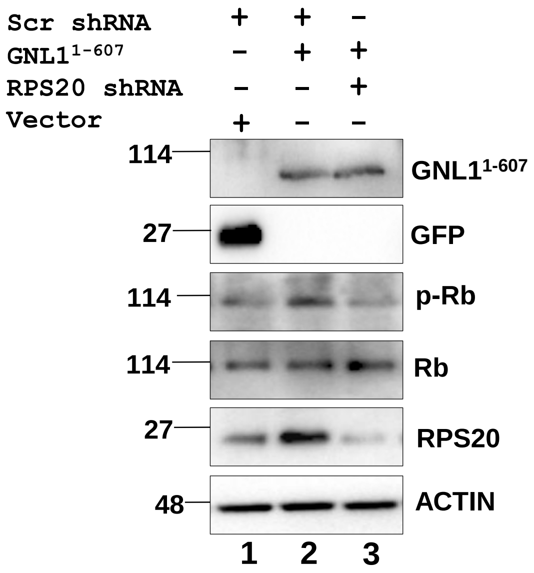

b)

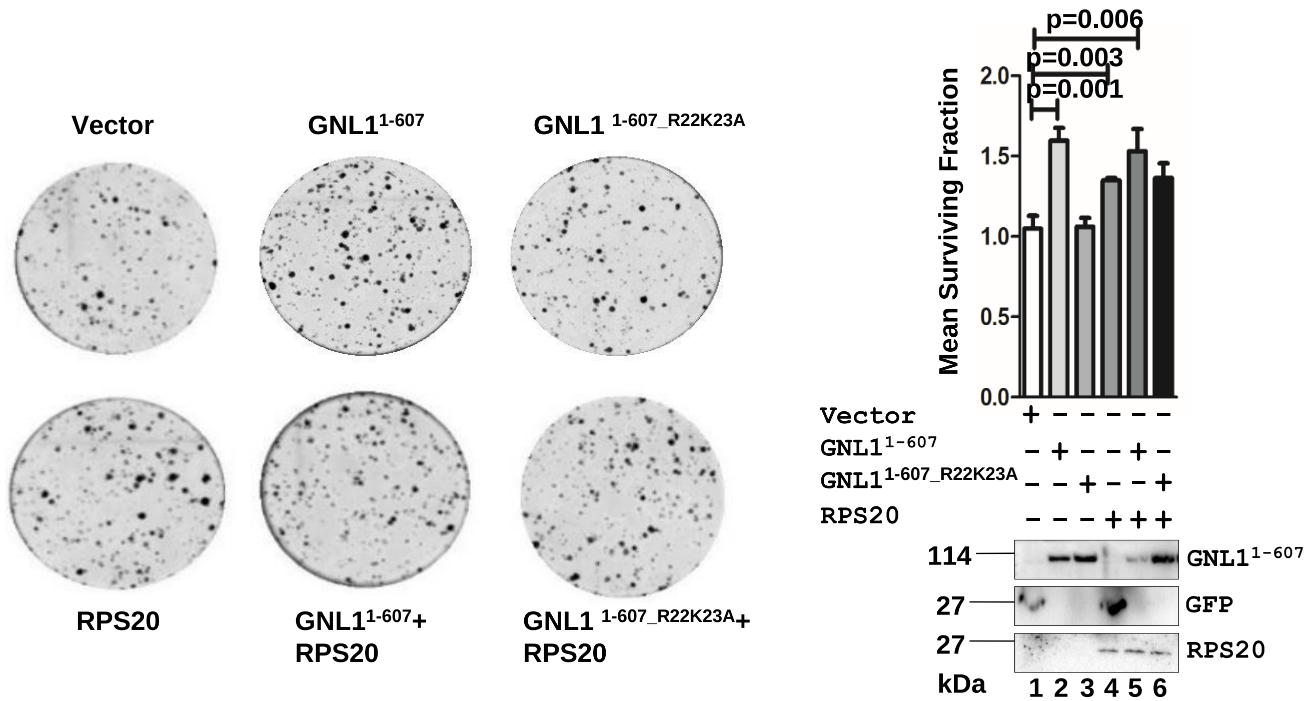

Supplementary figure 4: (a) Expression levels of phosphorylated and total Rb protein were determined by western blot analysis upon ectopic expression of GNL1$^{1\text{-}607}$ or control vector under RPS20 knockdown condition. Anti-phospho Rb(S780) and anti-Rb antibodies were used to detect the phospho and total Rb protein levels, respectively. The full length blots are presented in Supplementary figure 14. **(b)** GNL1 induced colony forming ability was checked by transfecting AGS cells with GNL1$^{1\text{–}607}$, GNL1$^{1\text{–}607_R22K23A}$, RPS20-FLAG or control vectors. After 48 hours of transfection, GFP positive cells were sorted and selected in culture medium containing G418 for 14 days. The mean surviving fraction of AGS cells was calculated and plotted as bar diagram as described in Materials and Methods.

Supplementary Figure 5

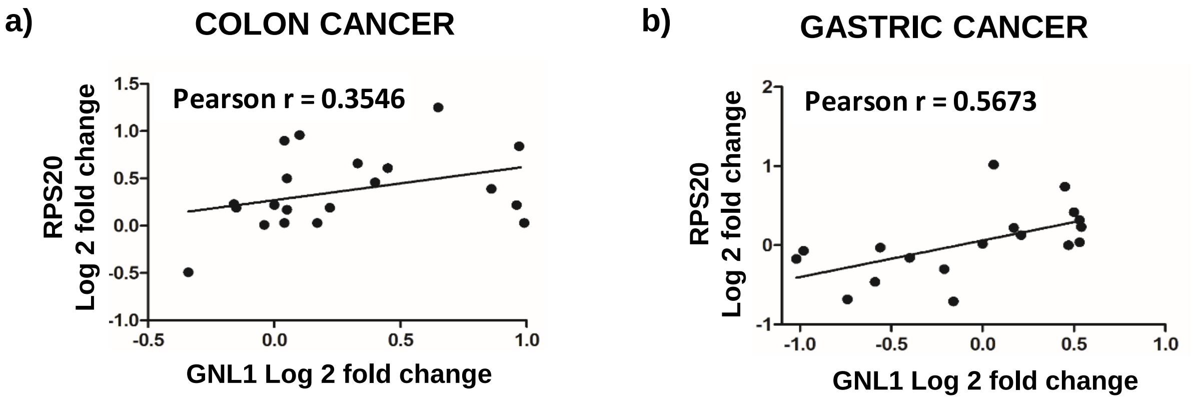

Supplementary figure 5: Evidence for the positive correlation of GNL1 and RPS20 expression in human cancers. Correlation analysis of GNL1 and RPS20 mRNA expression was calculated from the data available from BioXpress database for colon (Pearson r = 0.3546) **(a)** and gastric cancer tissues (Pearson r = 0.5673) **(b)**.

Supplementary Figure 6

Figure 1b

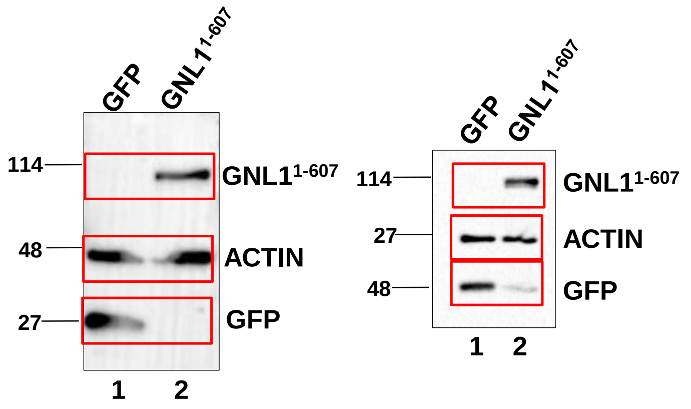

Figure 1c

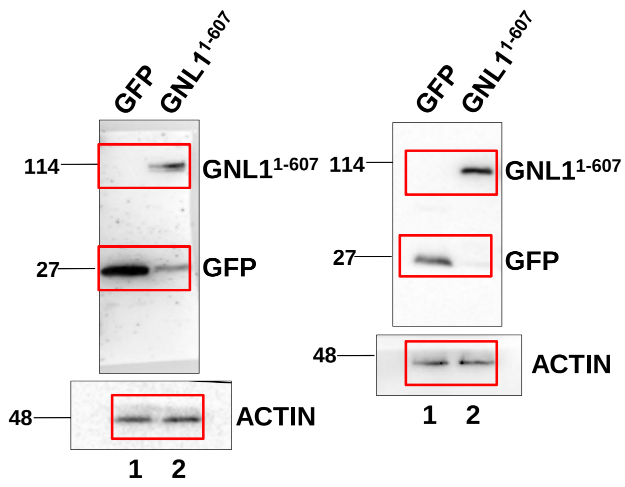

Figure 1d

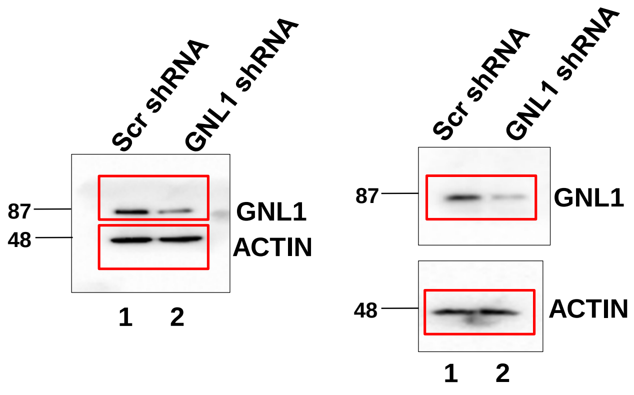

Figure 1e

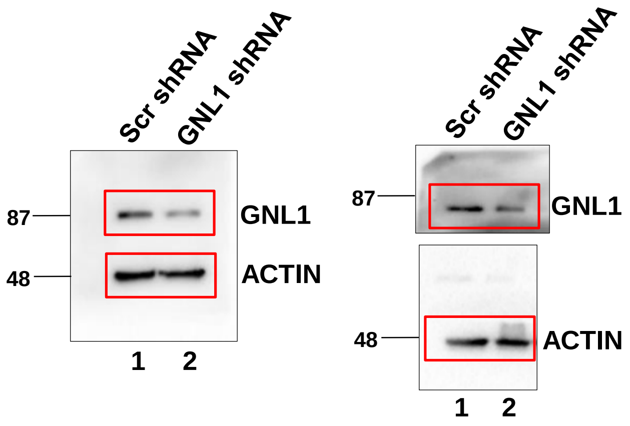

Supplementary figure 6: Uncropped western blot used in Figure 1

Supplementary Figure 7

Figure 2b

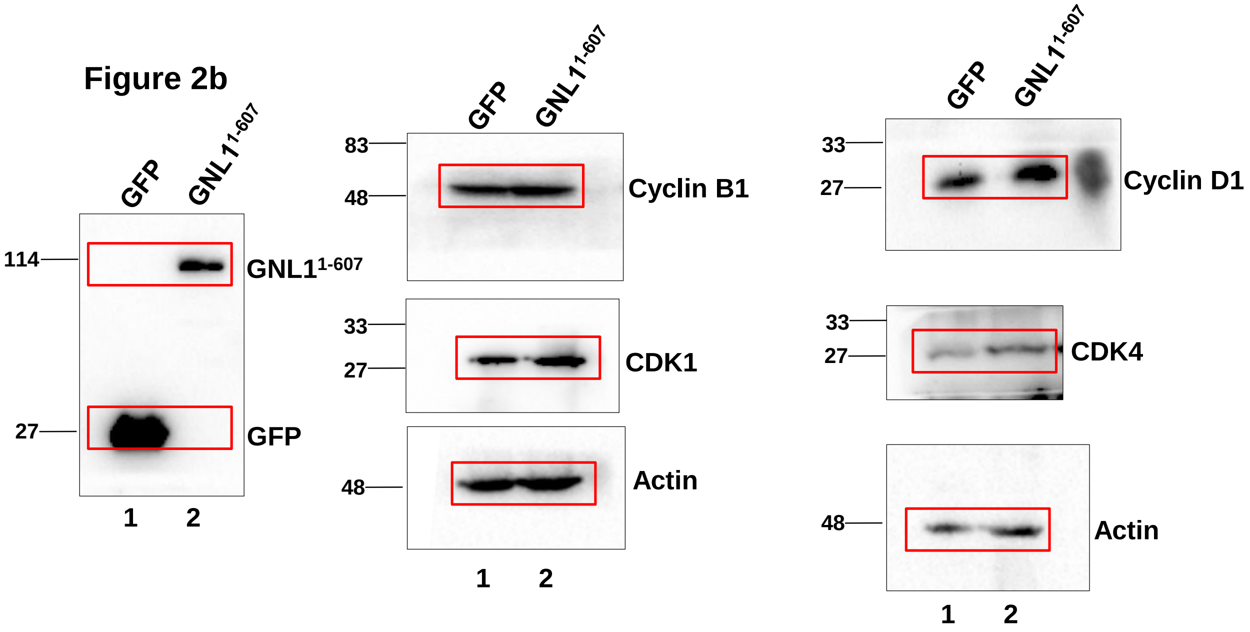

Figure 2c

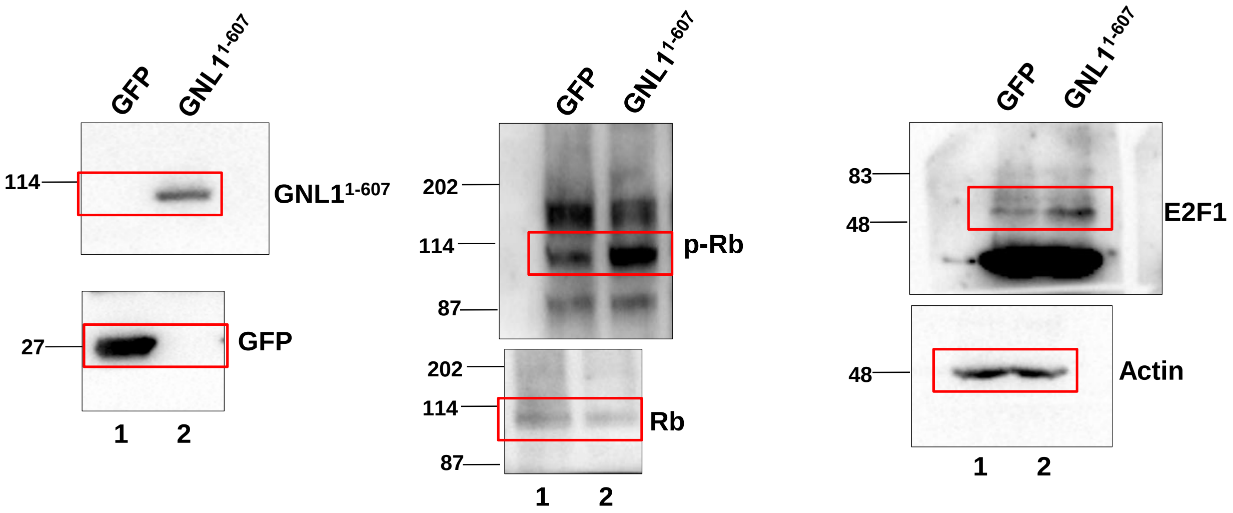

Supplementary figure 7: Uncropped western blot used in Figure 2b and 2c

Supplementary Figure 8

Figure 2f

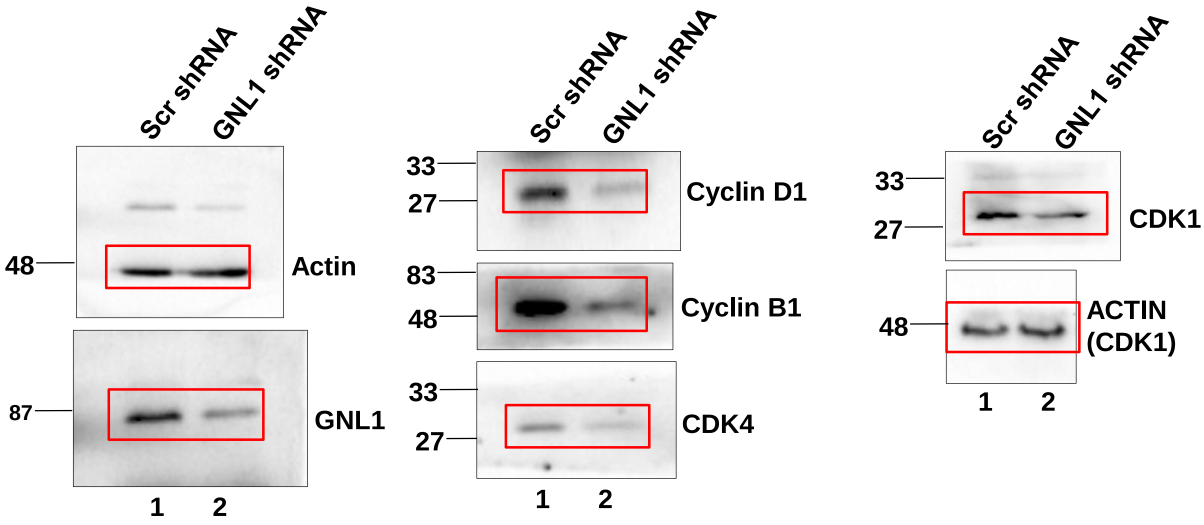

Figure 2g

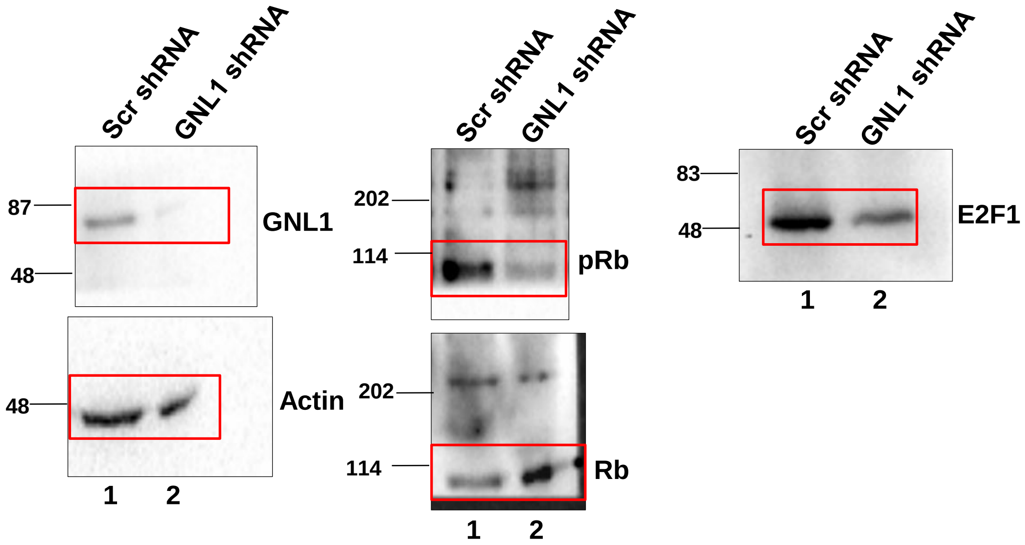

Supplementary figure 8: Uncropped western blot used in Figure 2f and 2g

Supplementary Figure 9

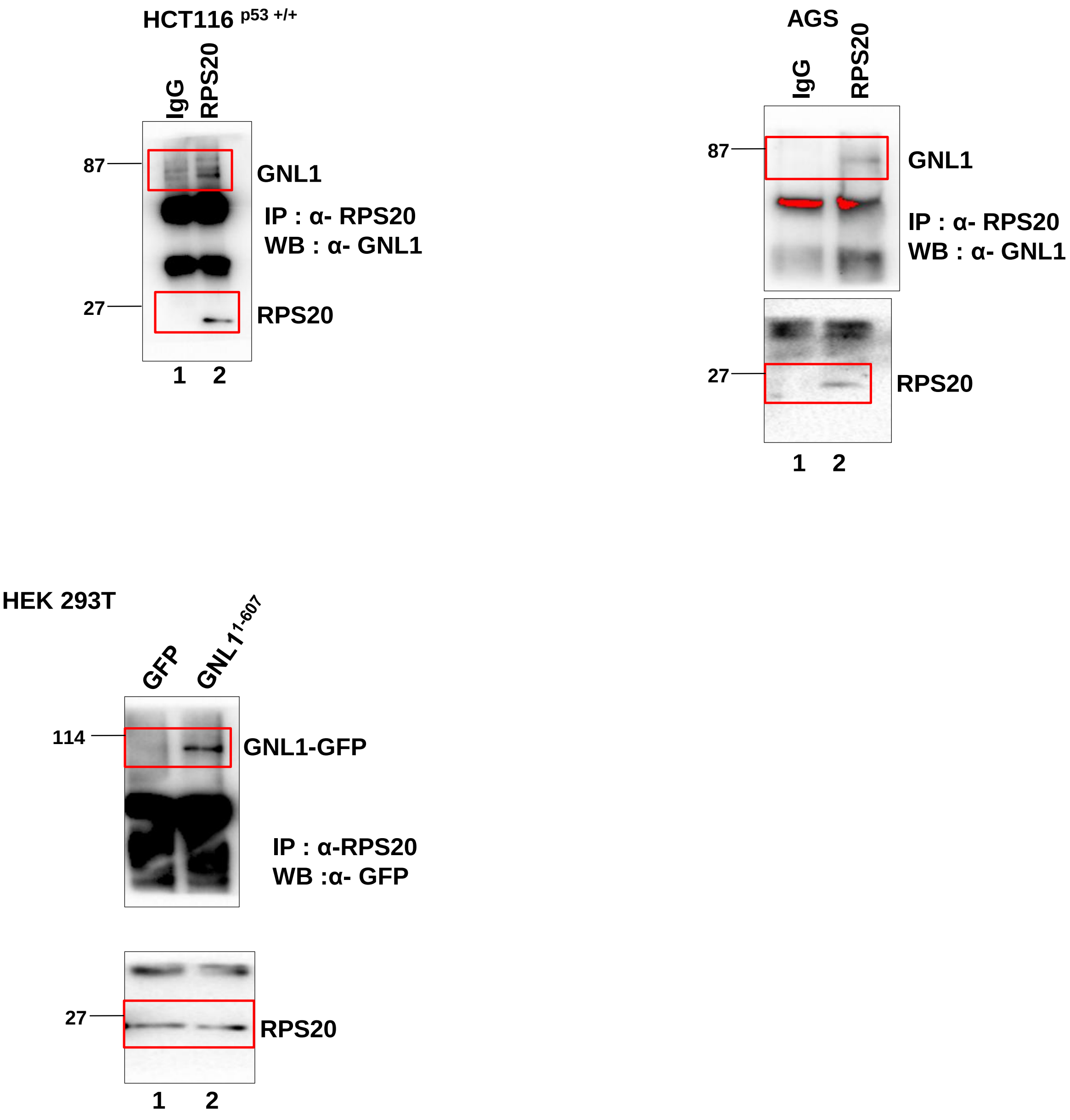

Supplementary figure 9: Uncropped western blot used in Figure 3d

Supplementary Figure 10

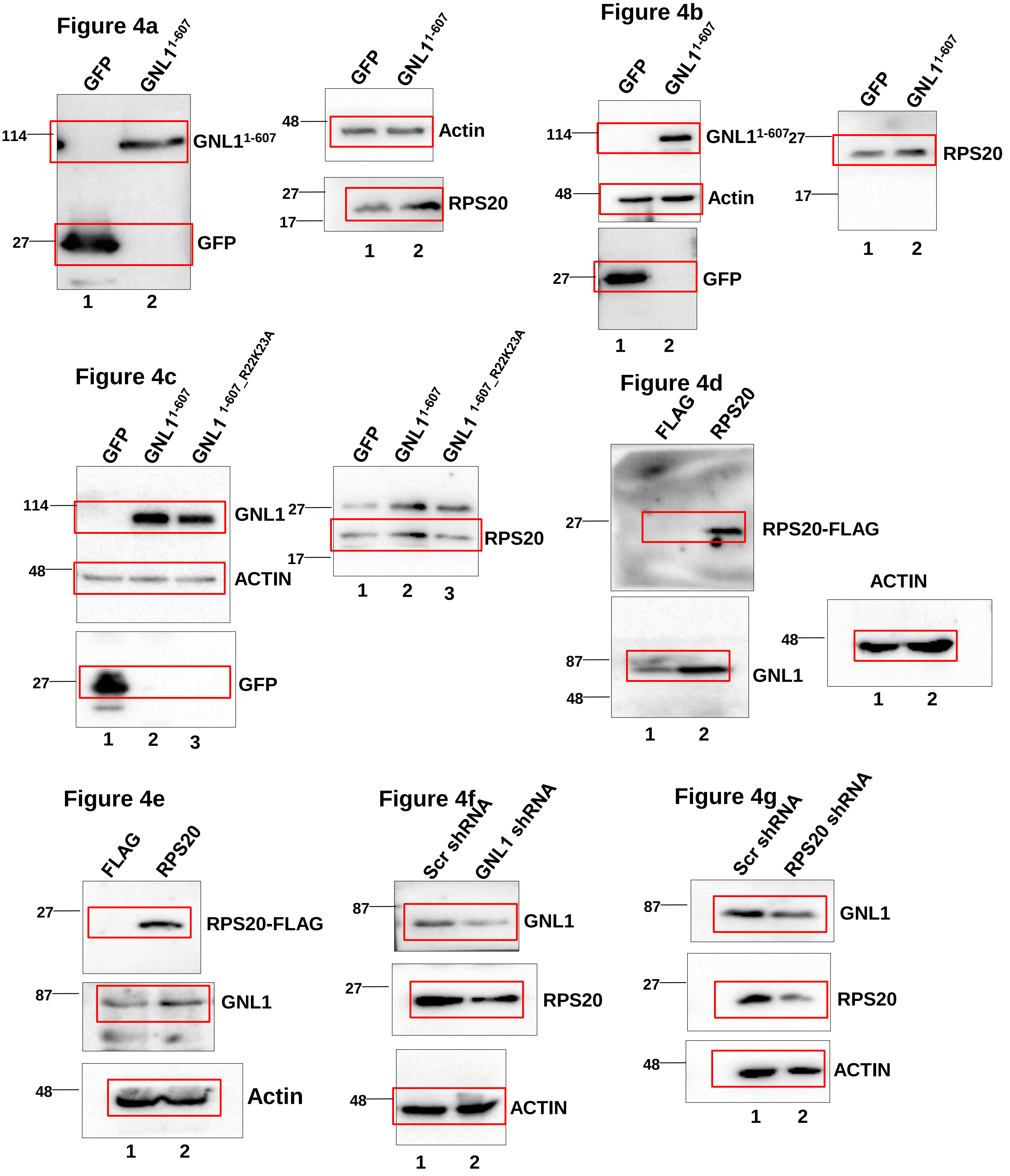

Supplementary figure 10: Uncropped western blot used in Figure 4

Supplementary Figure 11

Figure 5c

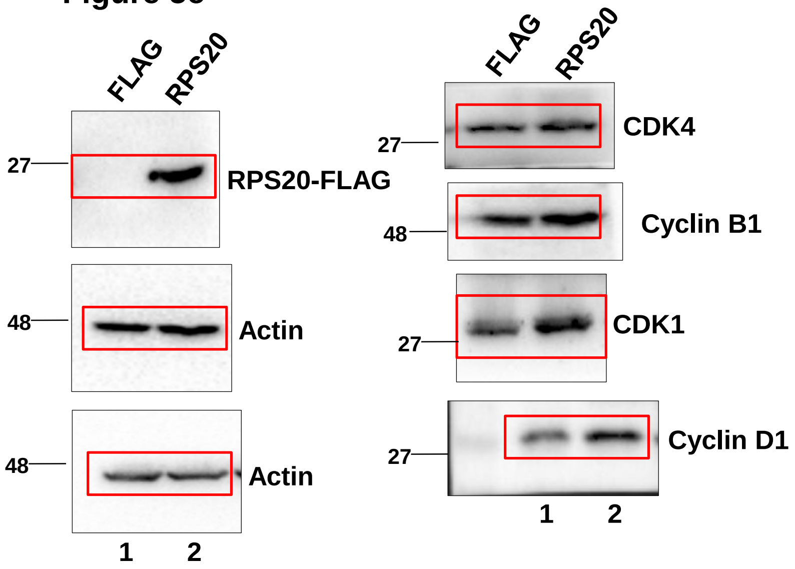

Figure 5d

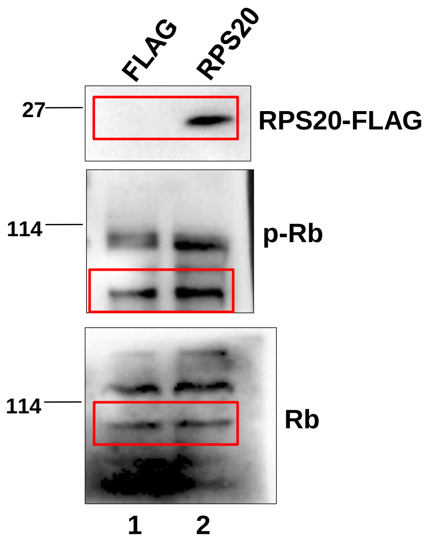

Figure 5h

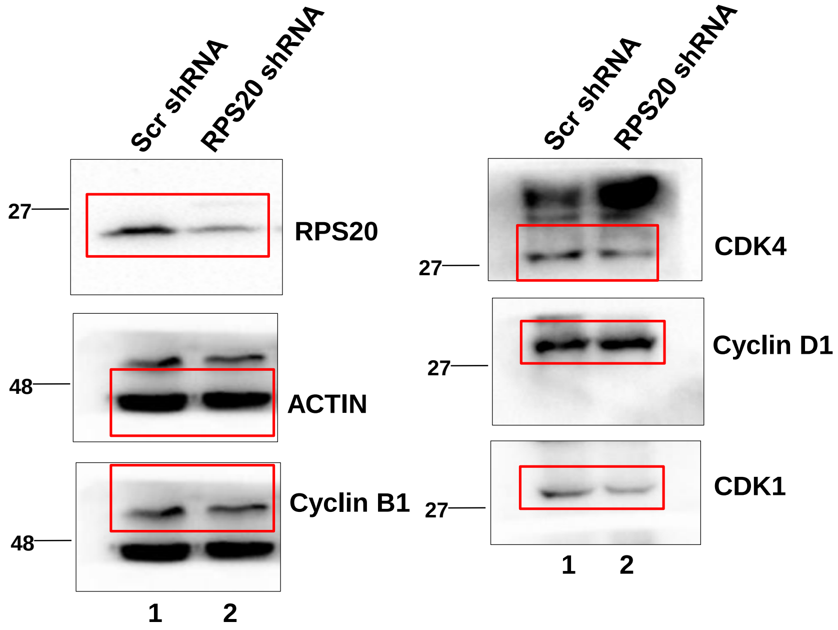

Figure 5i

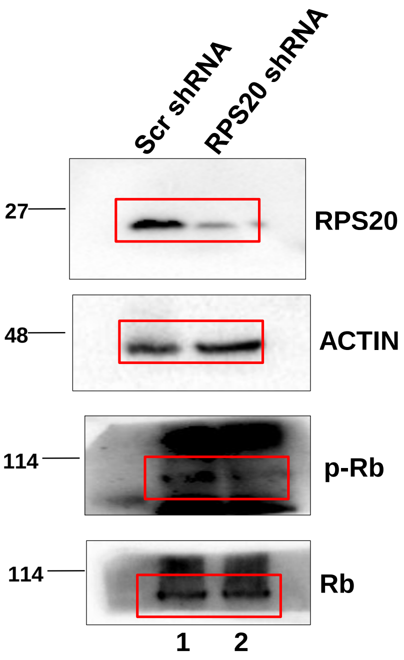

Supplementary figure 11: Uncropped western blot used in Figure 5.

Supplementary Figure 12

Figure 6b

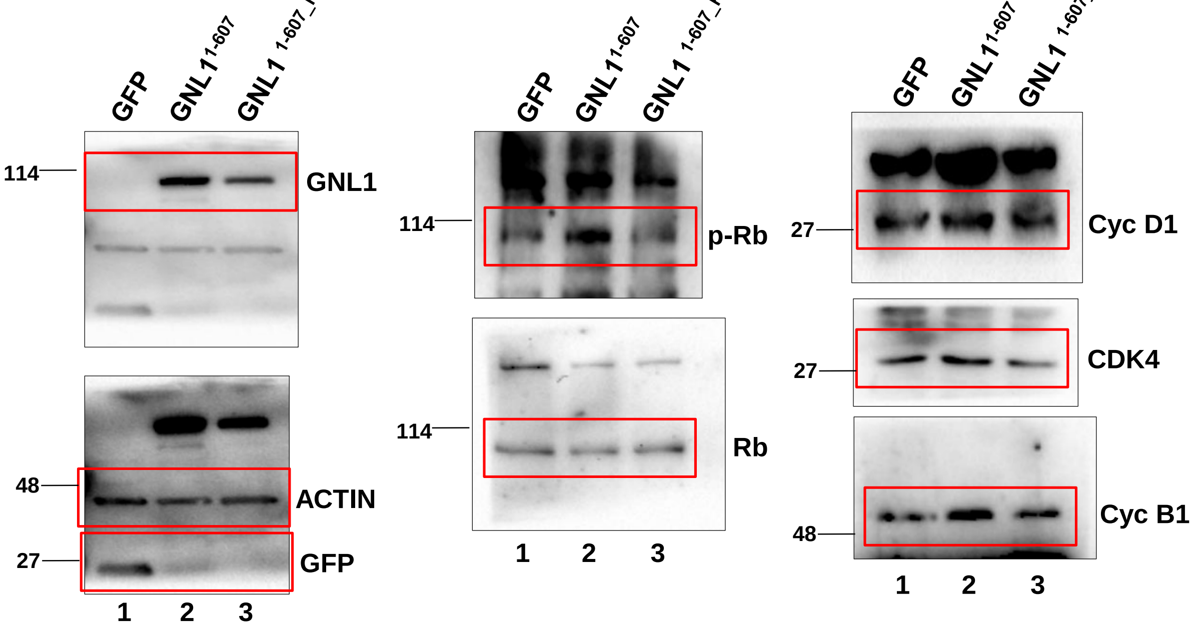

Figure 6c

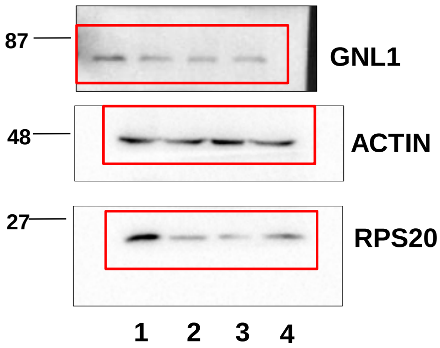

Figure 6d

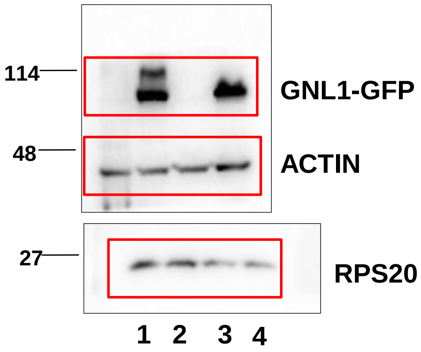

Figure 6e

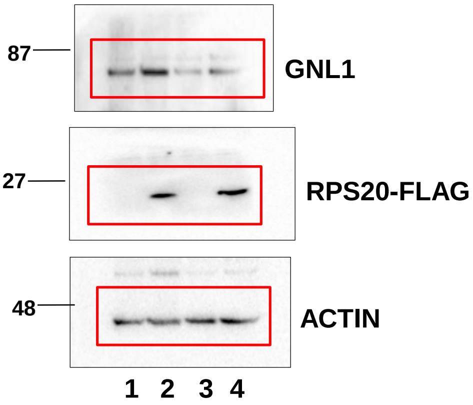

Supplementary figure 12: Uncropped western blot used in Figure 6

Supplementary Figure 13

Figure 7b

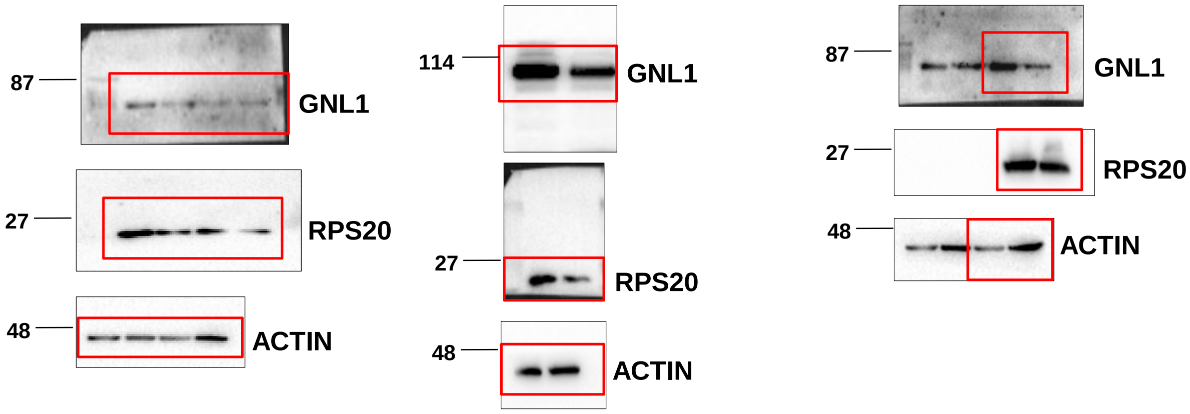

Supplementary Figure 3b

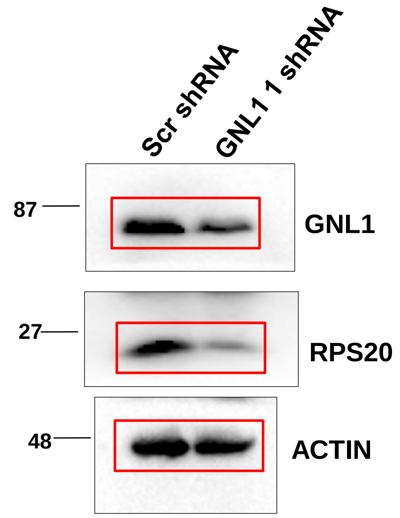

Supplementary Figure 3c

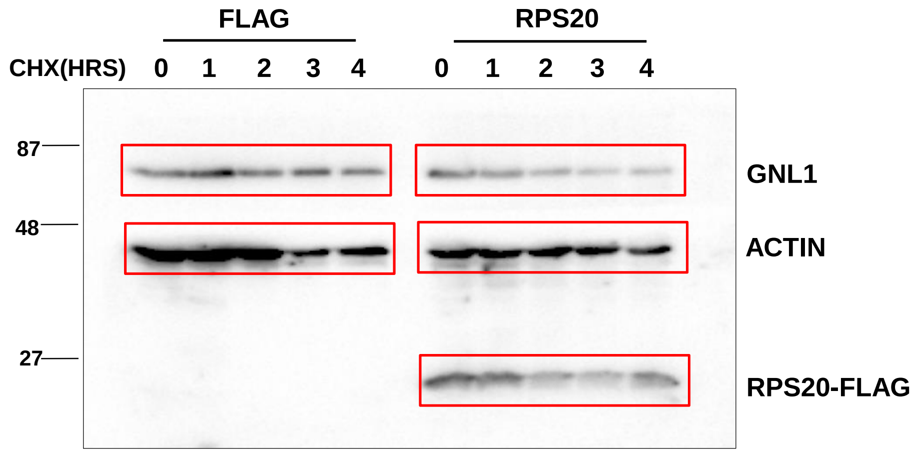

Supplementary figure 13: Uncropped western blot used in Figure 7b, Supplementary figure 3b and Supplementary figure 3c.

Supplementary Figure 14

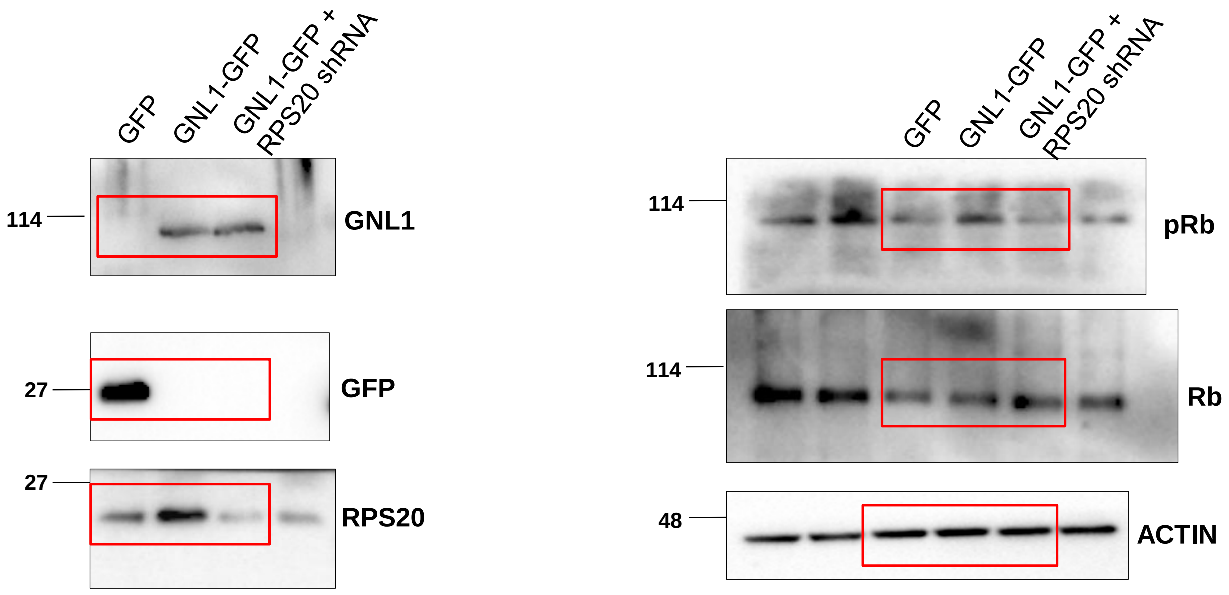

Supplementary figure 14: Uncropped western blot used in Supplementary figure 4a.
